# The Interplay between Interfacial Solvation and Surface Kinetics Tunes the Selectivity between Hydrogen Evolution and Zinc Electrodeposition

**DOI:** 10.1021/jacs.6c03962

**Published:** 2026-06-29

**Authors:** Daniel Escalera-López, Raquel Anastacio, Carlos Gomez Rodellar, Wiebke Frandsen, Sebastian Z. Oener, Beatriz Roldan Cuenya

**Affiliations:** Department of Interface Science, 28259 Fritz-Haber Institute of the Max Planck Society, Berlin 14195, Germany

## Abstract

Electrodeposition is a key technology for the fabrication of semiconductor interconnects, corrosion protection, decorative coatings, and even for chemical energy storage in batteries. Empirically discovered additives are regularly implemented in electroplating baths, but their impact on the electrodeposition mechanism, which involves the desolvation of metal ions across the electric-field-dependent double layer, has remained poorly understood. Herein, we perform overpotential-dependent Arrhenius analysis on Zn electrodeposition from strongly solvated zincate ([Zn­(OH)_4_]^2–^)-containing alkaline electrolytes in the absence and presence of the cationic polyquaternium-2 (PQ-2) electrolyte additive. We hypothesized that the water-soluble and positively charged PQ-2 can modulate the double-layer electrostatics, leading to distinct changes in the Arrhenius activation parameters. Without PQ-2, we observe that electrodeposition competes with hydrogen evolution, which is reflected in an extended region where an increasing (apparent) activation energy is overcompensated by an increasing Arrhenius prefactor. In contrast, when we add the positively charged PQ-2, we observe that the compensation region is suppressed and the Zn deposition kinetics proceed via efficient Butler–Volmer-type kinetics. The latter might arise due to charge inversion and a closer approach of the negatively charged zincate to the electrode surface that allows for electron transfer. Quartz crystal microbalance and electron microscopy support that the Butler–Volmer kinetics arise from a surface-controlled mechanism, which is linked to distinct morphological changes on the surface. These results are important to understand how the interfacial microenvironment can tune the activity and selectivity and how electrochemical kinetics can switch between solvation and surface-controlled regimes.

## Graphical abstract

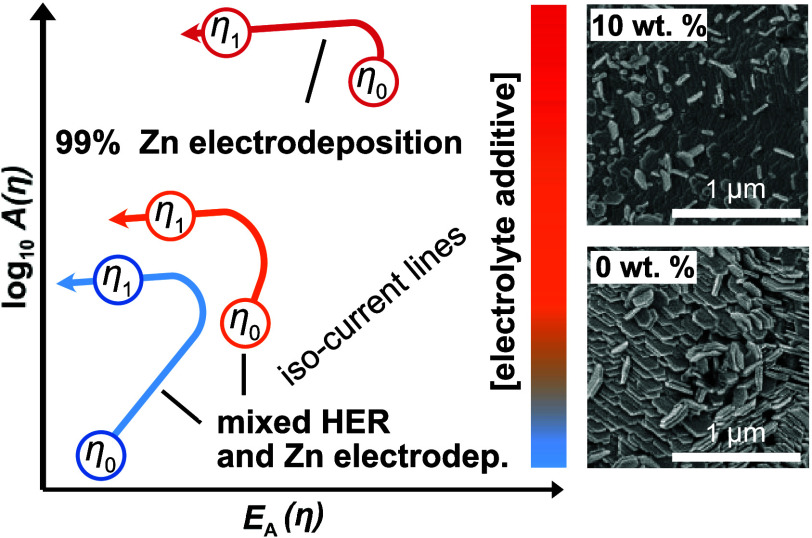

## Introduction

Electroplating stands as one of the widest and most relevant applications of electrochemistry in our daily life. Its covers corrosion-resistant coating manufacturing in automotive and aviation industries,^1^ metal interconnect plating on semiconductors^2^ and embellishing finishes for jewelry.^3^ During the charging cycle in Zinc-air batteries, metallic zinc is electrodeposited^4,5^ from alkaline electrolytes containing zincate anions, i.e., tetravalent OH-coordinated Zn^2+^ ions, [Zn­(OH)_4_]^2–^.^6–8^ This links battery storage capacity to the efficiency of Zn electroplating.^9–12^

To obtain such coatings, electroplating baths are carefully formulated, e.g., to maximize the faradaic efficiency for energy storage application or control the morphology of the deposits.^13–16^ The formulations generally include empirically discovered electrolyte additives, but fundamental understanding on the reaction kinetics in such environments is lacking. In particular, it is not clear how electrolyte additives impact the solvation kinetics of metal ion complexes.^17–19^ Despite explicit discussion of the importance of interfacial metal ion solvation in electrodeposition and corrosion by Gileadi and others,^20,21^ electron-centric kinetic pictures dominate the literature.

Recently, our team explored the impact of the electric-field dependent double layer on the interfacial solvation kinetics of protons and hydroxide species.^22–25^ More specifically, not only the apparent activation energy (*E_A_*), but also the Arrhenius pre-exponential factor (*A*) can depend on the applied overpotential and compensate each other, which is well-known for ionic charge transfer in the bulk^26^ and has been studied by Conway.^27^ However, we also discovered that changing kinetic regimes and log *A*-*E_A_* compensation can arise due to overpotential-dependent rate-limiting steps and degrees of rate control for the oxygen reduction reaction under high mass transport conditions.^28,29^ In fact, new microkinetic models^29–33^ suggest that such effects are also important for the hydrogen evolution reaction.^34,35^

Collectively, these results clearly show that electrochemical Arrhenius analysis can provide key insights into the overpotential-dependent kinetics, including in relation to interfacial excess charge and the electric-field dependent double layer,^22–25^ but that care must be taken in the interpretation, as well as exclusion of mass transport and other effects. In the context of electrodeposition, we hypothesized that (i) strongly solvated ions, such as [Zn­(OH)_4_]^2–^, might also be impacted by compensation effects and (ii) that water-soluble and positively charged quaternary ammonium polymers, such as polyquaternium-2 (PQ-2), that are regularly used to accelerate metal deposition,^36,37^ tune the interfacial microenvironment in very distinct ways.^38^ In particular, we speculated that the positively charged PQ-2 might partially compensate a negatively charged surface and the associated space charge region, potentially even leading to charge inversion^39,40^ and suppression of the associated compensation region. In general, understanding how ion solvation can be shaped is critical not only for enhanced electrodeposition, but also many electrocatalytic reactions, such as CO_(2)_ reduction to multicarbon products,^41–43^ let alone battery ion intercalation, geo- and biochemistry more broadly.

Here, we present electrochemical Arrhenius analysis in conjunction with electrochemical quartz crystal microbalance measurements on zinc electrodeposition and the competing hydrogen evolution reaction (HER) in alkaline electrolytes in the absence and the presence of PQ-2. We observe that the selectivity of Zn electrodeposition over hydrogen evolution can be tuned by promoting fast Butler–Volmer (surface) kinetics over slow log *A*-E*_A_*-compensation. These results provide fundamental insights in a field that is, so far, largely dominated by empirically discovered electrolyte formulations and, more broadly, highlight the importance of the local microenvironment in interfacial electrochemistry.^38^

## Results

To study HER and Zn deposition kinetics, temperature-dependent chronoamperometry (10–35 °C in 5 °C steps) was performed on pristine Zn foils in KOH (pH 13) in the presence or absence of dissolved ZnO ([ZnO] ≈ 1 mM) and with incremental PQ-2 contents from 0.1 to 15 wt %. The relatively low and narrow temperature range was chosen to ensure controlled conditions and reproducible results, as we observed irregular temperature-dependent polarization curves for longer times and higher temperatures. We hypothesize that these are caused by a contamination or larger Zn deposition rates leading to complex morphological changes that evade a systematic study. All data are based on steady-state currents at each individual potential (with the potential held for 60s) to mitigate the impact of pseudocapacitive discharging currents. The overpotential and temperature dependent currents were evaluated via Arrhenius analysis as described previously.^22,23^ The currents were limited to 5 mA cm^–2^ to minimize mass transport contributions in three-electrode configurations. Further Arrhenius plots with high linear regression values (*R* ^2^ > 0.95) were obtained. All Arrhenius plots and corresponding *R* ^2^ heatmaps are shown in Supporting Figures 1–4. All potentials are referenced vs the reversible hydrogen electrode (RHE), given the small temperature shift in the Zn standard redox potential (−0.99 mV 10 °C^1–^).^44^ As detailed in Supporting Note 1, the small temperature-dependent shift of the theoretically predicted open-circuit potential (∼ −2 mV 10 °C^–1^) was experimentally confirmed and neglected. eQCM measurements were carried out under temperature control using 5 MHz Au-coated quartz crystal electrodes. For details about preconditioning and calibration and electrochemical protocols, see the Methods section.

Figure 1a–d show temperature dependent polarization curves extracted from multistep chronoamperometry measurements with steady state currents (after holding for 60s) during the hydrogen evolution reaction on a metallic Zn foil in the absence of zincate, but with incrementally increasing PQ-2 content. The Zn foil is preconditioned with steady state cathodic currents. Initially, addition of small PQ-2 amounts (0.1 wt %) was found to yield suppressed HER activities (compare Figure 1a and b), reaching a minimum at 0.5 wt % PQ-2 (Supporting Figure 5). At higher PQ-2 contents, the HER activity increases again (Figure 1c), but recovers only partially at a PQ-2 concentration of 15 wt %. Previously, polycationic polymer additives were reported to modify the HER kinetics, although fundamental insights on the HER kinetics were not provided.^36,45^ In fact, when we evaluate the kinetics with differential Tafel analysis at 25 °C (Figure 1e–h), we observe high and nonlinear Tafel slopes even in potential and current windows where mass transport is not dominating the kinetics, especially for lower overpotentials and current densities (Figure 1b). Noteworthy, the three-electrode cells can support higher current densities, e.g., in absence of PQ-2 or 15% PQ-2, where we start to see a clear impact of mass transport limitations for current densities >4 mA cm^–2^ (Figure 1e). In general, challenged by corrosion/electrodeposition and passivation processes, the reported Tafel slopes for the HER on Zn vary rather widely in the literature with values around ≥120 mV dec^–1^ at lower KOH concentrations (≤1 M KOH), reflecting a Volmer-limited reaction step, and reaching substantially lower values at 10 M KOH.^46^

**Figure 1:**
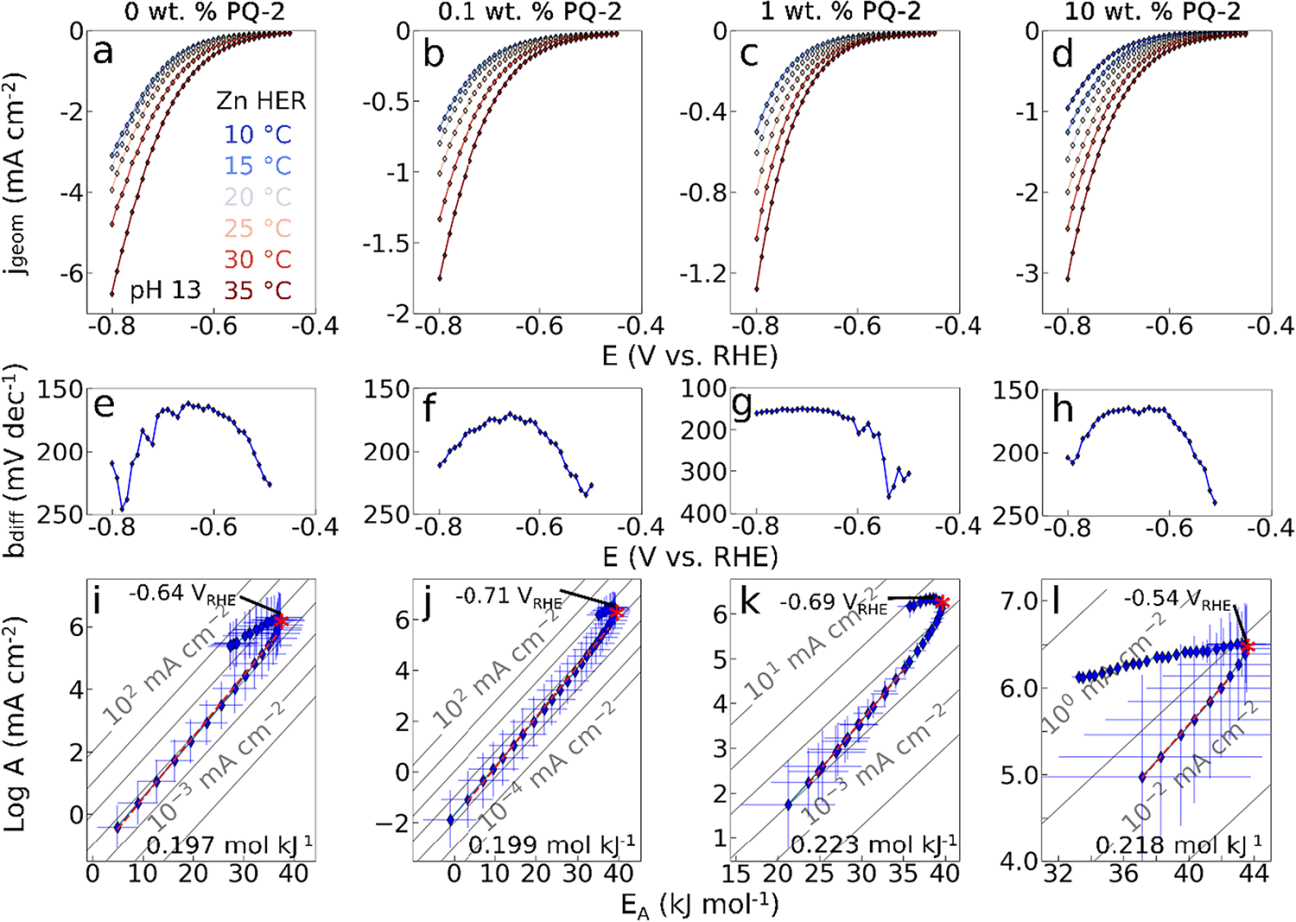
Polarization curves and potential-dependent Tafel slopes, pre-exponential factor and activation energy for the hydrogen evolution reaction in alkali aqueous electrolyte at specific polyquaternium-2 contents. (a–d) Representative current-averaged multistep chronoamperometric measurements at different temperatures (10–35 °C), (e–h) differential Tafel slope analysis at 25̊C, and (i–l) overpotential-dependent compensation plots [log *A*(η) vs *E_A_* (η)] obtained from Arrhenius analysis of multistep chronoamperometric measurements presented obtained in 0.1 M KOH electrolytes (pH 13) with respect to polyquaternium-2 (PQ-2) content (0, 0.1, 1, and 10 wt %). For all measurements, geometric current densities stemming from multistep chronoamperometric holds (potential was held during 60 s) are averaged for the last 15 data points (1.5 s). Kinetic maps (log *A*(η) vs *E_A_* (η)) plotted in 1­(i–l) are obtained by plotting the average values and standard deviations of the slope (*E_A_*) and origin intercept (log *A*) of the Arrhenius analysis per individual potential measured at five to six temperatures. Turning potentials are marked in 1­(i–l) as the potential with the highest *E_A_* (red asterisk). Linear regression *R* ^2^ values from Arrhenius analysis are above 0.95, see Supporting Figure 3.

Figure 1i–l shows the kinetic maps (log *A* (η), *E_A_* (η)) for the HER on metallic Zn foils with increasing PQ-2 contents. Similar to our recent results on the HER in acidic and alkaline hydrogen pump cells^34^ and across other reactions,^22–24,28^ we observe that *E_A_* (η) initially increases with overpotential, but is overcompensated by an increasing log *A*(η). At higher overpotentials, we observe again a distinct turning potential in the kinetics, where *E_A_* (η) and log *A*(η) change their behavior, which we observed, e.g., for the oxygen evolution^24^ and reduction reactions.^28^ With increasing PQ-2 content, we observe that the overpotential of the turning point first increases, in parallel to the reduced HER activity (Figure 1i–k). For 5–15 wt % PQ-2 the overpotential of the turning point is then reduced and the HER activity improves again (Figure 1l and Supporting Figure 5). Note that despite a changing PQ-2 content, the absolute values of the log *A*(η) and *E_A_* (η) are essentially the same as those of the pristine Zn foil. The main impact of PQ-2 is a change of the overpotential where these values are reached.

The similar apparent activation parameters with different PQ-2 contents mirror our findings for some OER catalysts^24^ and might imply an impact of the energetics of the electric-field dependent double layer, as also suggested by laser spectroscopic studies by Geiger and co-workers.^47,48^ In a conventional view, a surface-dominated PQ-2 (poisoning) process should lead to drastically changing activation energies. Similarly, whereas an overpotential-dependent intermediate coverage needs to be considered even for the seemingly simple HER,^29,31,49^ it is difficult to reconcile these activation parameter changes with an interfacial process that neglects the electric-field response in the double layer.

Mass transport effects might be implied in our data by the falling log *A*(η) in the Butler–Volmer region beyond the turning potential in Figure 1i–l and the nonlinear Tafel slopes beyond the plateau at intermediate potentials in Figure 1e–h. This could be caused by interfacial accumulation of H_2_ on the surface and active site blockage^50^ and is most clear for the data of the pristine Zn foil. However, for potentials below the plateau region (with a Tafel slope of ∼160 mVdec^–1^), we find larger and overpotential dependent Tafel slopes that arise from very well resolved (temperature) dependent polarization curves (Figure 1a–d). The traditional analysis of linear Tafel slopes excludes the nonlinear region at lower overpotentials, due to the impact of the reverse rate.^51^ Whereas at these large cathodic potentials we can neglect the fully suppressed hydrogen oxidation reaction, the Zn corrosion rate should be considered. However, when we compare the low overpotential range of the polarization curves in Figure 1a–d with the nonlinear Tafel slopes in Figure 1e–h, we find that the nonlinear Tafel regions do not coincide with linear polarization curves that would be signatures of a reversible range in Butler–Volmer kinetics.^51^ Thus, the impact of the reverse rate can be neglected (similarly to the HER on Au/C, for which the HOR does not occur^34^) and there is no reason to exclude the region at lower overpotentials from the analysis by only focusing on constant Tafel slopes at higher overpotentials.

In general, we caution of overly focusing on linear Tafel slopes when studying inner-sphere kinetics and relegating nonlinear Tafel slopes too readily to mass transport effects.^52^ In general, there is no fundamental reason for the assumption that only linear Tafel slopes inform on kinetic effects.^20,21^ As we have also shown previously even explicitly for high mass transport conditions,^28^ overpotential dependent Tafel slopes and charge transfer coefficients can originate from overpotential dependent rate-limiting steps and degrees of rate control.^53^ Furthermore, they might also be related to the interfacial solvation kinetics,^24,34^ where ion transfer coefficients do not have to be α = 0.5 nor take any specific discrete values that one might expect for multielectron transfer sequences.^20,21,54^ In fact, the transfer coefficients for (nominally) single step ion transfer will eventually become overpotential dependent.^20,21^ Arguably, even for electron transfer steps, overpotential-dependent Tafel slopes and transfer coefficients might arise, which is not captured in the phenomenological Butler–Volmer equation.^55–57^

### Temperature-Dependent Zinc Electrodeposition

For zincate-containing alkaline electrolytes in absence of PQ-2, we observe lower cathodic currents (Figure 2a) than in the absence of zincate (Figure 1a), consistent with previous reports.^8^ With increasing PQ-2 contents, the zincate-containing electrolytes show a similar change as the results in pure KOH. First, with increasing PQ-2 content we observe a decrease in the currents until 1 wt % (Figures 2b and Supporting Figure 6), followed by an increase in the currents beyond 5 wt % (Figure 2c). However, in contrast to the experiments in the absence of zincate, we observe an additional rise in currents for 15 wt % PQ-2 (Supporting Figure 7). This is strongly indicative of a higher rate of Zn electrodeposition. However, due to the competing HER that also proceeds in zincate-containing electrolytes, we later provide results from quartz-crystal microbalance experiments that disentangle their relative contributions. Finally, as shown in Supporting Figure 6-7, Tafel slopes at 25 °C show overall similar (overpotential) values as in Figure 1 with the exception for high PQ-2 contents, where the differential Tafel slope becomes almost constant. As detailed later, for the 10 wt % PQ-2 (Figure 2d,h) the kinetics are likely strongly dominated by the surface.

**Figure 2:**
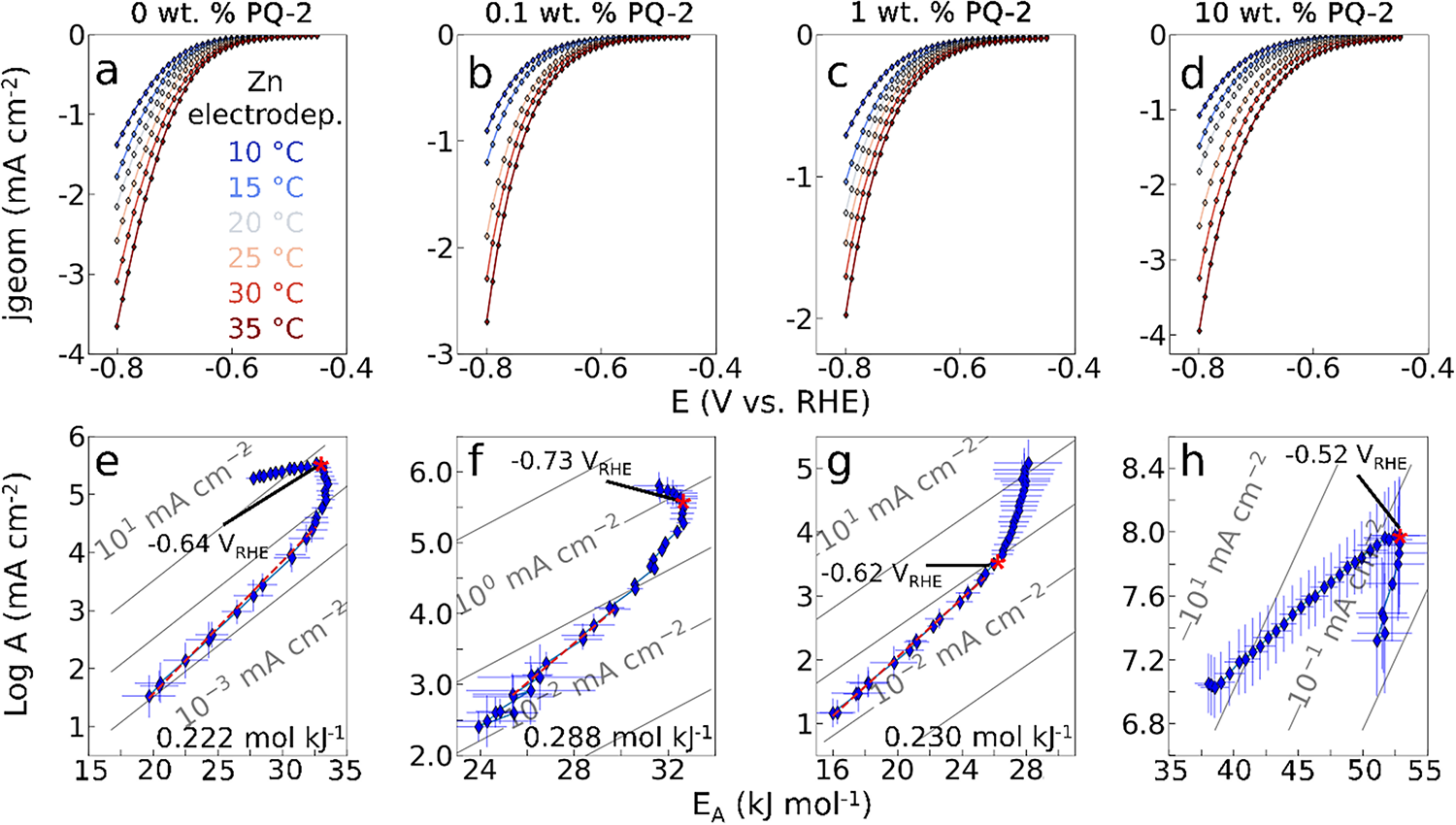
Polarization curves and potential-dependent pre-exponential factor and activation energy for zinc electrodeposition in alkali at specific polyquaternium-2 contents. (a–d) Representative current-averaged multistep chronoamperometric measurements at different temperatures (10–35 °C), (e–h) overpotential-dependent compensation plots [log *A*(η) vs *E_A_* (η)] obtained from Arrhenius analysis of multistep chronoamperometric measurements obtained in 0.1 M KOH electrolytes (pH 13) in the presence of dissolved zincate (1 mM ZnO) with respect to the polyquaternium-2 (PQ-2) content (0, 0.1, 1, and 10 wt %). For all measurements, geometric current densities stemming from multistep chronoamperometric holds (potential was held for 60 s) are averaged for the last 15 data points (1.5 s). Kinetic maps (log *A*(η) vs *E_A_* (η)) plotted in 2­(e–h) display the average values and standard deviations of the slope (*E_A_*) and origin intercept (log *A*) of the Arrhenius analysis per individual potential measured at five to six temperatures. Turning potential are marked in 2e–h as the potential with the highest *E_A_* (red asterisk). Linear regression *R* ^2^ values stemming from Arrhenius analysis are above 0.95, see Supporting Figure 4.

When we compare how the overpotential dependent activation parameters change with increasing PQ-2 contents (Figure 2e–g), we see a trend that is very similar to the ones for the hydrogen evolution reaction in Figure 1, suggesting that the HER is still dominating the kinetics at low PQ-2 contents. However, for the highest PQ-2 contents in Figure 2h, we observe that the absolute values of the activation parameters markedly deviate from the values at lower PQ-2 contents. Therefore, we chose to compare selected kinetic maps directly.

Figure 3a compares the HER kinetics in the absence and presence of zincate for 0 and 10 wt % PQ-2 and Supporting Figure 8 compares all PQ-2 concentrations. Figure 3b shows the maximum *E_A_* (η) and Supporting Figure 9 the maximum log *A*(η) to highlight the transitions. For the HER, all compensation plots overlay almost perfectly, with a reduction of the initial compensation regime with increasing PQ-2 content, as noted above. In the case of Zn electrodeposition (in the presence of ZnO), all kinetic maps initially also almost overlap. In absence of PQ-2, we find that the addition of ZnO leads to a shift toward lower iso-current lines, consistent with the lower overall currents in Figure 2 compared to Figure 2 for the lower PQ-2 contents. Below, we detail that even in absence of PQ-2 there is likely already Zn deposition occurring in parallel to the HER. For PQ-2 concentrations above 10 wt %, the apparent activation parameters with the Butler–Volmer regime shift toward higher currents and to substantially higher *E*_*A*_ (η) and *A* (η) values (Figure 3a and Supporting Figure 8b). Note that the rates increase for PQ-2 concentrations ≥ 10 wt % and for 15 wt % even surpass the values in the absence of PQ-2 (Supporting Figures 6–7). These results show that (i) increasing the concentration of cationic PQ-2 inside the double layer does not block transport through the diffuse or Stern layer, (ii) in general, the kinetics across different electrolyte formulations cannot solely be explained by single-step electron charge transfer and (iii) that mass transport effects are not causing the observed kinetic changes, as the cells can support higher current densities.

**Figure 3:**
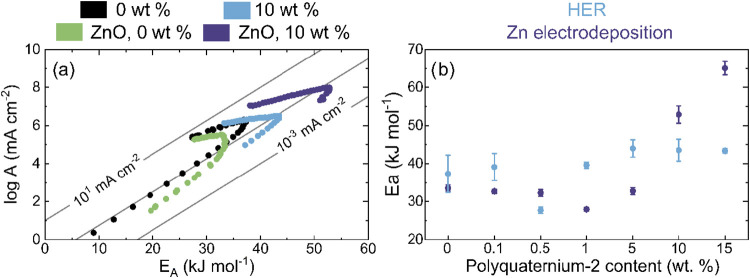
Comparison of kinetic maps and maximum activation energies obtained for the HER and zinc electrodeposition in alkali at specific polyquaternium-2 contents. (a) Kinetic maps [log *A*(η) vs *E*_*A*_ (η)] in the absence or presence of dissolved zincate (ZnO precursor) with respect to polyquaternium-2 (PQ-2) content (0 and 10 wt %). (b) Maximum activation energies extracted from kinetic maps in Figures 1–2 and Supporting Figures 5–7 in the absence (blue) or presence of dissolved zincate (1 mM ZnO, purple) with respect to the polyquaternium-2 (PQ-2) content (0 to 15 wt %). Error bars in Figure 3a are omitted for clarity (shown in Figures 1–2), while the data shown in Figure 3b correspond to the average values and standard deviations of the slope (*E*_*A*_) extracted from the Arrhenius analysis from Figures 1–2 and Supporting Figures 5–7. At the specific alkaline pH and ZnO concentrations employed, fully dissolved ZnO serves as the source of zincate.

A drastic upward change in the apparent activation parameters with increasing rates and improved product selectivity has been previously observed for highly concentrated NaClO_4_ electrolytes during CO electroreduction, where higher ionic strengths were linked to increasing Δ*S* values which aided the C–C coupling on Cu.^42,43^ Using *operando* Raman spectroscopy, the authors showed that high electrolyte salt concentrations (or water-in-salt electrolytes) can modulate the interfacial hydrogen bond network. Similarly, we postulate that the PQ-2 helps to preorganize the interfacial solvent in the absence and presence of zincate that speeds up the HER and Zn deposition. However, for Zn electrodeposition, another contribution arises, which might be due to a shift of the rate-limiting step, from a solvent mediated prestep to a surface-controlled Zn deposition step on certain crystal facets or defects.

To test whether the Zn deposition could also be tuned by concentrated salt electrolytes, we performed test experiments with 1 *m* (molal) NaClO_4_ (14 g per 100 g water) dissolved in 0.1 M KOH + 1 mM ZnO. As shown in Supporting Figure 10, we indeed observe qualitatively very similar changes, where the absolute activation parameters are substantially larger compared to the case without NaClO_4_ and PQ-2 (compare Figure 2e) and similar to the case of 10 wt % PQ-2 (compare Figure 2h). This suggests that the high ionic strength and Na^+^ concentration can impact the interfacial microenvironment and apparent Zn deposition kinetics, very similar to the case for CO electroreduction. However, it should be noted that the response is not identical to the case with PQ-2. Due to the larger prefactor, much larger currents are obtained in the case of 1 *m* NaClO_4_ and we observe branching, dendrite-like morphologies (Supporting Figure 10c), in contrast to the the morphology in presence of high PQ-2 content, as shown later. This suggests, that the PQ-2 might additionally modulate the interfacial electrostatic environment with specific functional groups, adsorption and diffusion moderation and templating of Zn growth. To explore the impact of specific functional groups and the polymer chemistry more extended studies will be needed in the future.

### Electrochemical Quart Crystal Microbalance and Faradaic Efficiency

To correlate the overpotential dependent activation parameters with the Zn electrodeposition directly, we performed electrochemical quartz crystal microbalance measurements (eQCM) on Zn thin films electroplated on Au-coated quartz crystals in the absence and presence of zincate and PQ-2. The electrochemical protocol is schematically depicted in Supporting Figure 11. For more details see the Methods section. To evaluate the impact of PQ-2 under quasi steady-state and transient operating conditions we performed slow cyclic voltammograms and on/off chronoamperograms. Besides the time-dependent geometric mass gain/losses (Δ*m*_geom_), charge-normalized integrated mass gains (*m*_integ_) stemming from the eQCM data are also presented to enable more meaningful comparisons.

Figure 4a shows the charge-normalized *m*_integ_ for slow (5 mV s^–1^) cyclic voltammograms in the presence of [Zn­(OH)_4_]^2–^ and supports that increasing PQ-2 contents yield increased Zn electrodeposition, as demonstrated by the 4-fold increased charge-normalized *m*_integ_ between 0 and 10 wt % PQ-2 (Figure 4a, bottom panel). Given the almost identical Δ*m*_geom_ response for each CV cycle, we conclude that the electrolyte is not being depleted by zincate. The effect of bare PQ-2 electroadsorption was accounted for by performing blank measurements in the absence of any zincate and with changing PQ-2 concentrations (Supporting Figure 12). Importantly, the mass gain for the zincate-free electrolyte is substantially lower than the case when zincate was present in the electrolyte (Figure 4). The on/off chronoamperograms yield similar conclusions. Regardless, the contribution of PQ-2 adsorption was accounted for in the calculation of the faradaic efficiency, as detailed in the Methods section. Importantly, at increasing PQ-2 contents in the zincate-free conditions, charge-normalized *m*_integ_ stemming from PQ-2 electroadsorption led to a plateau beyond 1 wt % PQ-2 content (Supporting Figure 12b, bottom panel). We hypothesize that this plateau originates from a full coverage of PQ-2 at the Zn-electrolyte interface. To test this, *in situ* characterization will be needed in the future, as the PQ-2 will desorb at open-circuit potentials and before transfer to ex-situ characterization, which necessitated the use of charged mica substrates in previous AFM studies on the PQ-2 morphology.^37^ This plateau of charge-normalized *m*_integ_ with increasing PQ-2 content also coincides with the shorter compensation regime in the HER kinetics in the absence of zincate in Figure 1 and Supporting Figure 5. In the presence of zincate, the rates increased when higher PQ-2 contents were present under transient operation (ca. 2-fold higher charge-normalized *m*_integ_ at 10 wt % vs 0 wt % PQ-2, see Supporting Figure 13b, bottom panel).

**Figure 4:**
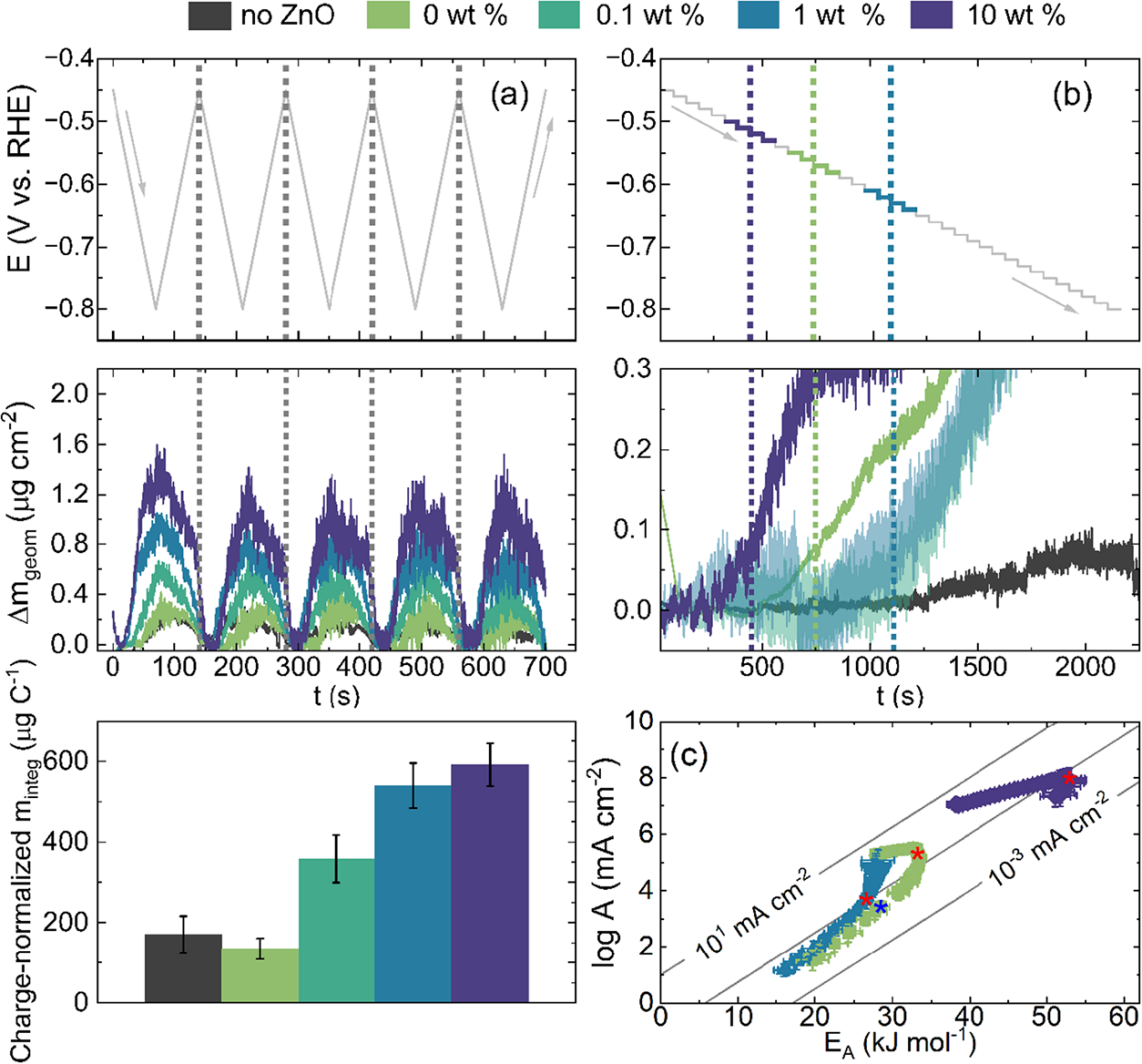
Quantification of mass gain and Zn electrodeposition with electrochemical quartz crystal microbalance (eQCM). (a) Time-dependent geometric mass gain (Δ*m*_geom_) and charge-normalized mass gain (m_integ_) obtained in the presence of dissolved zincate (1 mM ZnO) with respect to the PQ-2 content in the KOH electrolyte (pH 13) during sequential cyclic voltammetry acquisition (5 mVs^–1^). (b) Geometric mass gain (Δ*m*_geom_) obtained from multistep chronoamperometric holds on Zn-plated Au eQCM electrodes, zoomed to highlight Zn plating onset. The different experimental conditions need to be accounted for when comparing Δ*m*_geom_ in (a) and (b). (c) Kinetic map in the presence of dissolved zincate at specific polyquaternium-2 (PQ-2) contents (0, 1, and 10 wt %, right). The red star marks the turning potentials in the overpotential dependent activation parameters and coincides with the potentials marked with dotted lines in panel b above. The blue star marks the onset of deposition in absence of PQ-2. Note, the Zn plating faradaic efficiency is needed to comprehensively link the kinetic map to the Zn deposition.

To link the overpotential dependent activation parameters in the presence of zincate (Figure 2) with interfacial Zn mass gains, we performed multistep chronoamperometry at a representative temperature (25 °C), as shown in Figure 4b. Upon addition of [Zn­(OH)_4_]^2–^ in the electrolyte, the Δ*m*_geom_-t profiles, now governed by Zn electrodeposition, first shift to larger cathodic overpotentials between 0 and 1 wt % PQ-2 and then to lower overpotentials for 10 wt % PQ-2. We observe an overall positive potential shift (overpotential reduction) in the Zn deposition onset by 50 mV from −0.57 ± 0.02 *V*_RHE_ at 0 wt % PQ-2 to −0.52 ± 0.02 *V*_RHE_ at 10 wt % PQ-2. This shift in the activity of the zinc deposition is in good agreement with the turning point of the counterpart log *A*(η)–*E*_*A*_ (η) plots, as marked by the red stars in Figure 4c (see Supporting Figure 14 for *E*_*A*_ (η)) and the dashed lines in Figure 4b. However, whereas the 1 and 10 wt % PQ-2 show only negligible Zn deposition below the turning point, the 0 wt % shows already deposition at potentials that correspond approximately to the turning potential of the 10 wt % (∼ −0.53 *V*_RHE_, blue star in Figure 4c). This clearly shows that in absence of PQ-2 some of the Zn deposition can already occur in the compensation region. Overall, we conclude that Zn plating is kinetically favored in the Butler–Volmer regime beyond the turning point in the kinetic maps. The sooner this potential is reached, the faster the electrodeposition. However, to rigorously link the kinetics to the deposition, the Zn plating faradaic efficiency (FE) is needed.

Zn plating FE is extracted from charge-normalized *m*_integ_ vs faradaic charge passed for on–off (transient) and multistep chronoamperometry, as shown in Supporting Figure 15. For the calculation, see the Methods section. Increasing PQ-2 contents in on–off CAs steer Zn plating FEs from 10 to ∼96% at the highest PQ-2 content, mirroring the charge-normalized mass gain in Figure 4a. For the multistep experiments, the 0 wt % PQ-2 electrolyte yields a FE beyond 100%, which might indicate nonmetallic (presumably hydroxylated) Zn plating (potentially in the compensation region in Figure 4b). After an initial decrease in the FE with increasing PQ-2 up to 1 wt. PQ-2 (see increasing potential for Zn onset deposition shift in Figure 4b), the FE raises again and reaches another maximum (90 ± 30%) for 10 wt % PQ-2 (Butler–Volmer region in Figure 4b). The FE for the multistep experiments also follows the charge-normalized *m*_integ_ trends (Supporting Figure 13c).

To understand the difference between the FE from on–off CAs and multistep CAs, we focus on the clear difference between the two methods for 0 wt % (large difference) and 10 wt % PQ-2 (negligible difference) in Supporting Figure 15. First, for the on–off CAs there is no steady-state deposition inside the low potential (compensation) in Figure 4b, but the potential is immediately stepped to −0.8 *V*_RHE_. In contrast, for the multistep CA, there are steady-state deposition rates where each 10 mV step is held for 60s, allowing for substantial deposition in the compensation region for about 240s (green trace in Figure 4c). Conversely, the total FE in the low potential range is markedly different between multistep and on–off CAs. As noted, the >100% FE for the multistep CAs might indicate the formation of a hydroxylated Zn film. In contrast, for the on–off CAs the potential is suddenly stepped to the final potential of −0.8 *V*_RHE_ and the Zn deposition proceeds immediately in the Butler–Volmer region for 0 and 10 wt % PQ-2. As further discussed later, in the Butler–Volmer region the reaction proceeds over a different reaction pathway. Importantly, at −0.8 V_RHE_ the currents are substantially larger for 10 wt % than 0 wt % PQ-2 due to a substantial overpotential reduction (Figure 4b), which might induce mass transport limitations in the Butler–Volmer region for 10 wt %.

Mass transport effects are likely present even for nonpulsed electrodeposition given the eQCM configuration (lack of convection from bulk), as also apparent by the mass gain plateau beyond ca. −0.57 *V*_RHE_ for 10 wt % PQ-2 (ca. 750 s in Figure 4b). Mass-transport limited conditions (i.e., diffusion control) are generally linked with dendritic Zn growth^58,59^ (which is absent here), although factors such as bulk Zn concentration,^60^ plating electrolyte flow,^61^ or the presence additives^14,62,63^ can steer Zn plating toward other morphologies/stoichiometries. Noteworthy, our results indicate that the Zn deposition kinetics primarily adhere to Butler–Volmer kinetics at high PQ-2 content. Conversely, mass transport might impact the Butler–Volmer region here, e.g., by decreasing the prefactor with overpotential, but they cannot explain the log *A*(η)–*E*_A_ (η) compensation that we even observe in complete absence of any zincate and for pure HER currents. For the latter, we rule out a substantial impact of mass transport in the low current region that coincides with the compensation region, as detailed earlier in the discussion around the Tafel slopes in Figure 1.

### Scanning Electron Microscopy

Dissecting solvent from surface contributions is particularly challenging for electrodeposition, as the surface is continuously transformed during the reaction. Therefore, to evaluate the Zn electrodeposition morphologies we prepared Zn films potentiostatically (at −0.8 *V*_RHE_) at different thin film loadings (i.e., charge density cutoff) and imaged with scanning electron microscopy (Figure 5, Supporting Figures 16–18). As for the on–off CAs for the FE determination above, this approach steps immediately into the Butler–Volmer kinetic region, where deposition is sufficiently fast to provide enough material for clear SEM characterization.

**Figure 5:**
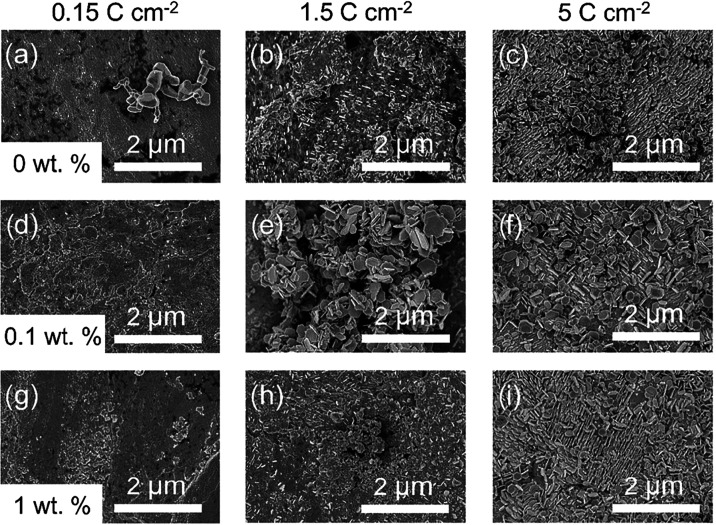
Evaluation of additive-dependent Zn electrodeposition morphology. SEM micrographs obtained for Zn film electrodeposits at charge density cutoff values ⟨σ_*q*_⟩ = −0.15, −1.5 and −5 C cm^–2^. Electrodeposits were prepared in 0.1 M KOH electrolytes (pH 13) with different polyquaternium-2 (PQ-2) contents: (a–c) 0 wt %, (d–f) 0.1 wt %, and (g–i) 1 wt %. SEM micrograph magnification: 25000X.

Without PQ-2, the electroplated Zn films present a layer-like, stratified morphology consisting of hexagonal-shaped nanoplatelets that increase in density with increasing charge densities (Figure 5a–c). The *strati* present no apparent preferential orientation growth plane, but interplanar voids across them can be observed (Supporting Figures 17–18). In addition, nanoplatelet aggregates with an off-plane growth profile were also identified. Importantly, increasing PQ-2 contents from 0.1 to 1 wt % does not drastically affect the layer-like morphology and primarily leads to a lower density of cavities (Figures 5d–i, Supporting Figures 18). This relative insensitivity to the presence of PQ-2 is consistent with the kinetic measurements, as reflected, e.g., by the relative insensitivity of the maximum activation energy in Figure 3b with increasing PQ-2 content up to 1 wt %. However, for larger PQ-2 content we see clear changes.

Figure 6a–d compare the film morphologies at the same charge density of 5 C cm^–2^, but with different PQ-2 contents between 0 and 10 wt %. As can be seen in Figure 6d and Supporting Figure 18, for 10 wt % PQ-2, the film becomes almost compact. For the kinetics in Figure 2–3, we see drastic changes in the Butler–Volmer region across PQ-2 concentrations. For 10 wt % PQ-2, the kinetics proceeds almost exclusively according to Butler–Volmer kinetics and the absolute activation parameters also increase substantially. This directly coincides with the formation of a much denser film in Figure 6d. These findings at high PQ-2 are also consistent with reports that ascribed quaternary ammonium compounds *leveling* properties to yield more uniform and compact Zn electrodeposits.^64,65^

**Figure 6:**
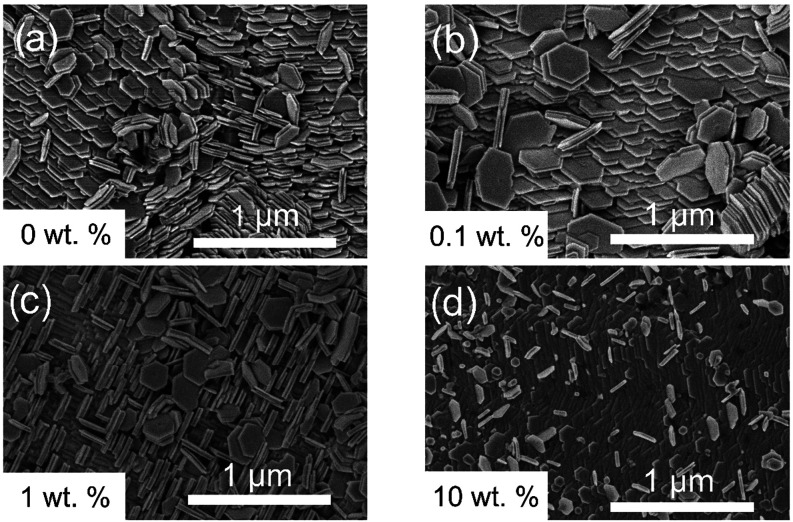
Evaluation of additive-dependent Zn electrodeposition morphology. SEM micrographs obtained for Zn film electrodeposits at charge density cutoff value ⟨σ_*q*_⟩ = −5 C cm^–2^. Electrodeposits were prepared in 0.1 M KOH electrolytes (pH 13) with different polyquaternium-2 (PQ-2) contents: (a) 0 wt %, (b) 0.1 wt %, (c) 1 wt %, and (d) 10 wt %. SEM micrograph magnification: 50000X.

For concentrations <10% PQ-2, we observe hexagonal-shaped nanoplatelets regardless of the absolute charge density cutoff value. However, the nanoplatelet morphology itself is unlikely responsible for the compensation region and the general trend in the activation parameters with changing HER vs Zn electrodeposition selectivity. As detailed above, we are only sampling the final deposition in the Butler–Volmer region with the SEM characterization and the compensation region being even present in the absence of any Zn electrodeposition and the changes of the activation parameters between 0 and 1 wt % PQ-2 evade a classical surface-controlled kinetic picture. In conclusion, PQ-2 showcases a multifaceted effect in accelerating Zn electrodeposition from the interfacial solvation kinetics standpoint but also steering toward enhanced thin film homogeneity by tuning the growth profile analogously to other shape-controlling electrolyte agents.^66^ Our results also evidence that especially for kinetics that are proceeding in a Butler–Volmer region, surface structural and chemical changes are critical.

## Discussion

Approaches to understand electrodeposition kinetics have classically relied on a quasi-steady-state overlapping hemispherical diffusion model proposed by Scharifker and Hills during potentiostatic transients,^67^ presenting intrinsic limitations.^68,69^ At longer time scales, kinetic analyses often stems from analysis of linear Tafel slopes at a single temperature,^52,70,71^ which is intrinsically challenged to capture ion transfer and solvation kinetics^22,24,34^ in the electric-field dependent double layer. In general, across the literature there is a large tendency to neglect the activated nature of the interfacial ion transfer and solvation kinetics and relegate the latter to regular mass transport limitations. In fact, despite pioneering experiments^20,21^ and theory^72,73^ on the importance of metal ion solvation kinetics, the literature remains focused on electron transfer kinetics. This might be at least in part due to the success of theory and experiments^74,75^ to solve “the enigma of metal ion deposition”,^20^ i.e., why Ag^+^ electrodeposition is one of the fastest electrochemical reactions. The Ag^+^ is thought to get very close to the electrode surface without losing its hydration shell, enabling strong orbital interactions between the electrode and solvated ion,^74^ which enables fast Butler–Volmer type kinetics.^75^ This situation is similar to the fast HER kinetics on the Pt group metals in acid and the strong d-band overlap with the 1s orbital of the solvated H^+^.^76^

Divalent metal ions such as Zn^2+^ have a very large hydration shell in acid, let alone the zincate ([Zn­(OH)_4_]^2–^) in alkaline conditions. In general, this leads to much larger hydration energies and their close approach to the surface, which is required to induce the first electron reduction step,^72^ becomes energetically demanding. Here, for the Zn electrodeposition from zincate in 0–1 wt % PQ-2, we observe a kinetically unfavorable compensation region, where Zn deposition rates are low and also some HER is proceeding in parallel. This suggests that in this region, the (electric-field dependent) interfacial environment is not able to selectively extract zinc ions from the electrolyte and requires a substantial overpotential. In contrast, for PQ-2 concentrations 10 ≥ wt %, we observe Butler–Volmer kinetics for the Zn deposition (Figures 2–3) with near constant Tafel slopes (Supporting Figure 6h). This might suggest a rate-limiting electron transfer step,^52^ that becomes feasible to reduce the Zn^2+^ to Zn^+^, because the positively charged polycationic PQ-2 might help draw the negatively charged [Zn­(OH)_4_]^2–^ closer to the surface within the scope of charge inversion,^40^ present in multiple biological platforms^77–80^ but also at solid-electrolyte electrical double layers for multivalent electrolytes.^39^ Additionally, during mass transport-limited long-term electrodeposition, PQ-2 might act as a buffering layer to prevent inhomogeneous Zn nuclei growth ascribed to dendritic electrodeposits.^45^

Once the [Zn­(OH)_4_]^2–^ can come closer to the surface with the help of PQ-2 and the reaction is limited by an electron transfer dominated Butler–Volmer regime, the surface structure and chemistry become critical. In fact, we observe a clear change in the surface morphology, as we transition out of the *A*(η)–*E*_A_ (η) compensation region that is associated with mixed HER and Zn electrodeposition currents and occurrence of nanoplatelets to a pure Butler–Volmer type region at PQ-2 ≥ 10%, associated with pure and dense Zn electrodeposition. The importance of a surface dominated regime is also further supported by the drastic change in the absolute values of the apparent activation parameters for Zn electrodeposition with PQ-2 ≥ wt. 10%. The large increase of the prefactor and activation energy strongly suggests that the apparent activation parameters are now dominated by a new rate-limiting step that is related to the Zn^+^ reduction and nucleation on specific surface motifs,^37,72^ which might further evolve during the reaction. In other words, by speeding up the solvation prestep, the Zn surface can tune the selectivity of the reaction by preferentially depositing Zn over the HER, primarily based on surface energetic considerations and largely unhindered by the impact of the solvation inside the double layer. In this picture, the selectivity is intrinsic to the surface chemistry and structure and the solvation prestep rather acts as suppressor for the desired selectivity.

The kinetics in this study were obtained via multistep steady-state chronoamperometry and are not dominated by mass transport effects, as further detailed below. Conversely, even in the compensation region and at low PQ-2 contents, where we hypothesize about a solvation barrier for [Zn­(OH)_4_]^2–^, the surface structure evolves with time, leading to continuous growth of nanoplatelets. This might suggest an important link between the solvation kinetics inside the local double layer structure at dynamically evolving edge and corner sites of the nanoplatelets. At higher PQ-2 contents, we observe a leveling of the surface morphology and concomitant Butler–Volmer kinetics and a more homogeneous Zn growth across the whole surface. Conversely, the question arises whether other processes, such as the nucleation or crystal growth might dominate the overpotential-dependent activation parameters at shorter times, including in the overpotential range of the compensation region. Resolving such processes will require future operando microscopy and time-resolved Arrhenius analysis with a μs-s time resolution, which was recently introduced by us.^23^

The transition between the compensation region (nanoplatelet formation) at lower current densities and the Butler–Volmer region (dense film formation) at higher current densities is unlikely caused by mass transport effects. As detailed above, the compensation regions occur already at low current densities, and substantially before mass transport suppresses the prefactor and causes nonlinear Tafel slopes after the plateaus of constant Tafel slopes in Figure 1e–h. Further, it even occurs for the HER only. Moreover, with the addition of positively charged PQ-2 that deposits onto the surface and thereby blocks part of the mass transport, well-resolved Butler–Volmer kinetics appear over a larger range with constant Tafel slopes (Supporting Figure 6h). In other words, the PQ-2 does not sufficiently block mass transport to prevent Butler–Volmer kinetics, but causes a decreasing prefactor at higher current densities. Finally, to understand the kinetic effect of the adsorbed PQ-2 on the activation parameters comprehensively, changes in the heat capacity with the adsorbed PQ-2 need to be considered and would require future *in situ* microcalorimetry.^81–83^ Importantly, the general relevance of our findings even beyond electrodeposition become further evident when we compare the kinetic maps to altogether different reaction conditions.

The trends in our kinetic maps in Figure 3 are very similar to the ones reported by Hall and co-workers for CO reduction on Cu^42,43^ to C_2_H_2_ as a function of the NaClO_4_ concentration and studied with operando Raman spectroscopy. For lower electrolyte concentration ([NaClO_4_] < 1 m), the team observed overpotential dependent water ordering that coincided with a compensation between the pre-exponential factor and the activation energy.^43^ However, for higher electrolyte concentration, the initial compensation region was suppressed and Butler–Volmer kinetics were induced almost over the whole overpotential range, which coincided with substantially enhanced current densities and FE for C_2_H_2_ formation. Importantly, the pre-exponential factor and activation energy increased substantially in absolute terms. The team explained the latter enhancement of the prefactor with an increased disorder in the interfacial water. However, as detailed above and discussed by some of us previously,^43^ this second increase of the log *A* - *E*_A_ values might originate from a shift of a rate-limiting (solvent-dominated) transition state to a surface-dominated transition state that is now dominated by the C–C coupling step on the surface, where structural and chemical *operando* changes are critical to understand the activation parameters. Strikingly, when we performed Zn electrodeposition control experiments with 1 *m* NaClO_4_ (Supporting Figure 10), we observed a very similar qualitative behavior as reported by Hall and co-workers for CO electroreduction, where the absolute activation parameters increased compared to the case without NaClO_4_. This strongly suggests a shared interfacial solvent effect for CO reduction and Zn electrodeposition. At the same time, for the Zn electrodeposition, we observe distinct morphological changes between the 1 *m* NaClO_4_ and the ≥ 10 wt % PQ-2, strongly suggesting another important effect of PQ-2. We hypothesize, that the distinct PQ-2 polymer chemistry modulates and mediates the Zn deposition, leading to leveling growth and reduced deposition rates, compared to the higher current for 1m NaClO_4_, quickly leading to dendritic structures. This raises the promise to utilize tailored polymers not only for electrodeposition, but to control microenvironments for selective CO_2_ reduction and other electrocatalytic reactions.

## Conclusion

In conclusion, we demonstrate how charged electrolyte additives can tune the interfacial microenvironment within the double layer and modulate the apparent activation parameters derived from Arrhenius analysis. The observed shift in selectivity between the hydrogen evolution reaction (HER) and Zn electrodeposition is associated with pronounced changes in the kinetic maps, likely reflecting a transition in the rate-limiting states. Overall, our findings highlight that electrochemical Arrhenius analysis provides valuable insights, not only into electrocatalytic kinetics, but also into electrodeposition on dynamically evolving surfaces.

## Methods

### Electrochemical Setup and Temperature-Dependent Measurements

Electrochemical measurements were carried out using a Gamry Reference 3000 potentiostat (Gamry Instruments, USA) in a three-electrode configuration using a Nalgene cell (Ø ∼ 6 cm, 125 mL). The Zn working electrodes (WE) were obtained after mechanically cutting a Zn foil (0.25 mm thick, 99.98 ≥ metals basis, Thermo Fischer Scientific) into square pieces of 2.5 × 2.5 cm, and individually inserted into a leak-tight PEEK sample holder (geometric area = 0.385 cm^2^, Redoxme AB, Sweden). For each measurement, a new Zn foil was employed. The Zn foils were ultrasonically cleaned sequentially in isopropanol and DI water to remove organic traces prior to the electrochemical measurements. The reference (RE) and counter electrode (CE) consisted of a leakless RHE reference electrode (MiniHydroflex, Gaskatel GmbH, Germany) and a 5 mm diameter glassy carbon rod (HTW GmbH, Germany), respectively. A custom-machined PTFE holder was employed to ensure a systematic relative position between RE/WE/CE and seal the Nalgene cell. All cell parts in direct contact with the electrolyte were sequentially cleaned to remove organic/inorganic traces before any measurement as follows: overnight soaking in KMnO_4_, followed by piranha solution immersion and rinsing in ultrapure water (Elga PURELAB, resistivity 18.2 MΩ cm, TOC < 1 ppb). An additional boiling step in ultrapure water was carried out for PTFE, glass and PEEK parts.

The employed electrolytes were deoxygenated with Ar bubbling (Linde, 5N purity) before and during the measurements to mitigate oxygen entrainment, while the cell was placed in a double-walled jacketed bath controlled by a Julabo CORIO CD-800F refrigerated/heating circulator. Temperature-dependent Arrhenius analysis was carried out by acquiring multistep chronoamperometries at temperatures between 10 and 35 °C in 5 °C intervals, monitoring the cell temperature with a PTFE-coated K-type thermocouple. The alkaline electrolytes employed consisted of 0.1 M KOH (potassium hydroxide monohydrate, semiconductor grade, Sigma-Aldrich) with incremental contents of polyquaternium-2 ranging from 0.1 to 15 wt % (62 wt % in water, Sigma-Aldrich), in the presence/absence of 1 mM ZnO (nanopowder, 99% metal basis, Alfa Aesar). This concentration yields, at the selected electrolyte (pH 13), close-to-saturation conditions.^84^ For the polymer-free high-salt control, an otherwise identical 0.1 M KOH + 1 mM ZnO electrolyte was prepared with an additional 14.05 g sodium perchlorate monohydrate (NaClO_4_·H_2_O, Supelco/Merck, LiChropur, 99.0–101.0%) per 100.00 g Milli-Q water, yielding a nominal 1 *m* (molal) NaClO_4_ electrolyte. To avoid oxidation, all electrolytes were injected into the electrochemical cell under potential control (−0.75 *V*_RHE_, 240 s).

The Zn electrode was conditioned (a schematic is shown in Supporting Figure 11) by cathodic potentiodynamic cycling (−0.85 to −1.1 *V*_RHE_, 100 cycles, 100 mVs^–1^) (Supporting Figure 19) and a potentiostatic hold at the most reductive potential applied for 15–30 min, followed by the multistep chronoamperometric holds (held for 60 s, 10 mV voltage step size) (Supporting Figure 20). All plots stemming from these measurements are obtained by plotting the steady-state currents at each individual potential hold (Supporting Figure 20) and averaging over the last 15 data points that correspond to the last 1.5 s of each 60 s potential to mitigate the impact of pseudocapacitive discharging or reduction currents in the analysis, where Arrhenius analysis was performed as described elsewhere.^22,23^ The potentiostat was set to maintain potential control between electrochemical steps and temperatures to prevent incursions to open circuit potential which would compromise Zn stability. All potential values reported are expressed with respect to the RHE scale, given the almost-negligible temperature shift in the Zn standard redox potential, as theoretically predicted^44^ to be −0.99 mV/10 °C and experimentally found to be −2 mV/10 °C (Supporting Figure 21).

### Electrochemical Quartz Crystal Microbalance Measurements (eQCM)

eQCM measurements were carried out using a Gamry eQCM 15 M temperature-controlled cell (Gamry Instruments, USA) coupled with a eQCM 10 M module, employing 5 MHz Au-coated quartz crystal electrodes (Part Nr. 971–00051, geometric area = 0.95 cm^2^, overlapping area = 0.21 cm^2^) under temperature control (Julabo CORIO CD-800F circulator). The eQCM electrodes were calibrated daily by monitoring potentiodynamic Cu plating and stripping in an electrolyte comprising 5 mM CuSO_4_ (copper sulfate pentahydrate, 99.999% trace metal basis, Sigma-Alrich) in 1 M H_2_SO_4_ (sulfuric acid, Suprapur, 96% v/v, Merck). Five plating/stripping cycles were recorded between −0.095 and 0.455 *V*_RHE_ at 50 mV s^–1^, whereby the Sauerbrey constant (*C*_*f*_) was estimated using the slope of the eQCM resonant frequency change (Δ*f*) versus the total charge passed (*C*) during Cu plating using the following expression

$$C_{f}=\frac{{\mathrm{Slope}}_{\Delta f\text{−}C}\times F\times A_{\mathrm{geom}}\times n}{M_{\mathrm{Cu}}\times 10^{6}}$$

where Slope_Δ*f*–*C*_ corresponds to the Δ*f*–*C* slope (in Hz C^1–^), *F* the Faraday constant, *A*_geom_ the eQCM geometric area (in cm^2^), *n* the number of electrons transferred during Cu plating/stripping (assumed to be 2 e^–^/Cu atom) and *M*_Cu_ the molecular mass of Cu. The daily calibrated *C*_*f*_ were experimentally determined to be 50–75 Hz cm^2^ μg^–1^, close to the theoretical value of 56.6 Hz cm^2^ μg^–1^. The Sauerbrey equation, which determines the relationship between Δ*f* and the experimental mass change (Δ*m*) can be therefore simplified as follows

$$\Delta f=-C_{f}\Delta m$$

After Cu plating/stripping, the Au eQCM electrode was electrochemically cleaned by cycling between 0.6 to 1.85 *V*_RHE_ in 3% HNO_3_ at 500 mV s^–1^ until a steady-state voltammogram was obtained (ca. 50 cycles). A leakless RHE and a Pt mesh CE were employed in both cases.

To perform analogous measurements in the eQCM setup, a Zn thin film was freshly prepared by pulsed electrodeposition with a target Zn loading of 500 μg_Zn_ cm^–2^ (assuming 100% Faradaic efficiency): galvanostatic pulses between −50 and 0 mA cm^–2^ were exerted in a 20/5 ms on/off fashion until the targeted loading was achieved (1476 cycles). The resulting thin films only lead to a 0.5–0.8% shift of the quartz crystal initial resonant frequency, and therefore, the Sauerbrey relationship can be quantitatively employed.^85^ During the initial Zn plating, a Hg/HgO RE (internal electrolyte: 1 M KOH, RE-61 AP, ALS Inc., Japan) and a 5 mm diameter glassy carbon rod CE were used, along with a 30% KOH electrolyte (ca. 6.9M) saturated with ZnO (ca. 0.7 M). This solution was solely used for the initial plating. Subsequently, to remove any saturated Zn electrolyte traces in between different experiments, both eQCM PEEK body and O-rings were cleaned by soaking in KMnO_4_ and piranha solution, respectively, followed by boiling/rinsing with copious amounts of ultrapure water. Once the cell ware cleaning was completed, the eQCM measurements that followed were all performed in 0.1 M KOH with varying PQ-2 contents for the blank HER measurements as well as for Zn electrodeposition measurements (1 mM ZnO added to the aforementioned electrolytes).

Besides slow cyclic voltammograms and on/off chronoamperometric measurements at the electrochemical window of interest, multistep chronoamperometric holds were recorded in identical conditions as those of the Arrhenius analysis at 25 °C (for parameters, see Supporting Figure 11). A selected set of electrolytes in the presence/absence of ZnO were used, with varying PQ-2 contents: 0, 0.1, 1, and 10 wt %. During all HER/Zn electrodeposition measurements, a leakless RHE and a 5 mm diameter glassy carbon rod CE were used.

Zn plating faradaic efficiencies (FEs) during on–off and multistep chronoamperometric holds are calculated by converting the integrated mass gains during Zn plating (*m*_gain,Zn_) to equivalent charge passed (*Q*_Zn,integ_), assuming a 2-electron plating process yielding metallic Zn. Before equivalent charge conversions are calculated, average blank integrated mass gains (*m*_gain,blank_) are subtracted per equivalent electrolyte employed (Supporting Figure 11), as PQ-2 electroadsorption and electrolyte ions adsorption are foreseen to contribute to *m*_gain,Zn_. The resulting FE are calculated, using Faraday’s law of electrolysis, as follows

$$\mathrm{FE}=\frac{Q_{\mathrm{Zn},\mathrm{integ}}}{Q_{\mathrm{tot}}}=\frac{\frac{\left(m_{\mathrm{gain},\mathrm{Zn}}-m_{\mathrm{gain},\mathrm{blank}}\right)\times 10^{-6}\times z\times F}{M}}{Q_{\mathrm{tot}}}\times 100$$

where *z* stands for the number of electrons exchanged during the redox process (2 for metallic Zn plating), *F* for the Faraday constant (96485 C mol^–1^), *M* for Zn molecular weight (65.39 g mol^–1^), and *Q*_tot_ for the integrated charge passed during chronoamperometric measurements. Both *m*_gain,Zn_ and *m*_gain,blank_ are expressed in μg, and obtained after averaging no less than two independent eQCM measurements.

Measurements were also carried out in the as-received Au-coated quartz crystal electrodes, using the same experimental parameters for on/off chronoamperometic measurements shown in Supporting Figure 11 and a wider electrochemical window during cyclic voltammetry (0 to −0.8 *V*_RHE_, 5 mV s^–1^, 3 cycles). Electrochemical cleaning in between PQ-2 containing electrolyte measurements was carried out in a 3% HNO_3_ electrolyte (ca. 0.5 M), at an electrochemical window between 0.6 to 1.85 *V*_RHE_. Transient CAs presented comparable trends to those previously observed (Supporting Figures 22–23). However, unlike the Zn-coated electrodes, two additional voltammetric peaks could be found in the Δ*m*_geom_ -t profiles in the presence of [Zn­(OH)_4_]^2–^ and PQ-2. The first peak (here found at ca. −0.3 *V*_RHE_ during the CV forward scans) was previously ascribed to the electro-adsorption of the zincate-loaded polymer, directly followed by the overpotential-driven mass uptake ascribed to Zn electrodeposition along with a proposed polymer compacting-readsorption.^36^ Voltage reversal in the backward scan yields an additional redox feature which, as no electrodeposition hysteresis loops are present, would be indicative of [Zn­(OH)_4_]^2–^ ion transport limitations instead of surface roughening or PQ-2 limited-transport to the electrode.^86^ In addition, Zn electrodeposition reached a peak at lower PQ-2 contents (1 wt %) based on charge-normalized *m*_integ_ values, where higher polymer contents are detrimental to the performance. Steady-state voltammograms obtained after electrochemical cleaning in 3% HNO_3_, recorded at an oxidative window slightly beyond the Burshtein minimum (Supporting Figure 24),^87^ also showcase distinct changes in polycrystalline Au redox features. Prior exposure to increasing PQ-2 contents not only shifts the Au(111) facet oxidation feature to upward potential values (from 1.55 to 1.65 *V*_RHE_), but also seems to steer polycrystalline Au reconstruction toward higher abundance of the Au(100) facet (peak at ca. 1.35 *V*_RHE_).^88,89^ We therefore conclude, similarly to underpotential deposition phenomena, that PQ-2 undergoes metal (facet)-specific interactions with Au which affect its intrinsic electrochemistry, but also yield improved Zn electrodeposition kinetics at lower PQ-2 coverages.

### Morphology Characterization: Scanning Electron Microscopy (SEM)

Zn thin film samples were prepared in the same setup used for the temperature-dependent measurements, with the exception of a ca. 5 × 1.5 cm Zn foil used as CE. Films of varying thickness were prepared by potentiostatic chronocoulometry at −0.8 *V*_RHE_ using charge density cut-offs ⟨σ_*q*_⟩ of −0.15, −1.5 and −5 C cm^–2^. In order to monitor the thin film morphology, SEM micrographs were acquired with a Hitachi S-4800 ultrahigh-resolution SEM at 1.5 kV acceleration voltages using the secondary electron detector (working distance: 4 mm).

### Arrhenius Analysis

The analysis of temperature-dependent measurements is performed by fitting the log_10_ of the experimental current densities (log_10_ *j*) against the inverse of temperature for every overpotential (η) following the linearized form of the Arrhenius equation

$$\log_{10}j\left(\eta \right)=\log_{10}A\left(\eta \right)-\frac{E_{A}\left(\eta \right)}{\ln10R}\frac{1}{T}$$

where *R* is the ideal gas constant, *T* is the temperature in K, ln 10 is the natural log of 10, log_10_ *A* is the log 10 of the pre-exponential factor, and *E*_*A*_ is the activation energy. The origin intercept from the fit corresponds to log_10_ *A*. To obtain *E*_*A*_, the slope of the fit is multiplied by—ln 10*R*. The linear fitting is done with an ordinary least-squares estimator.

### Tafel Analysis

The differential Tafel analysis is performed by calculating the numerical gradient of the overpotential (η) vs the log_10_ of the current density at a given temperature for every data point

$$b_{\mathrm{diff}}={\left(\frac{\partial \eta }{\partial \log_{10}j}\right)}_{T}$$

Numerically, this is done by computing the second order accurate central differences in the interior data points and the accurate one-sides (forward or backward) at the boundaries. This is a common method to calculate the gradient of a data array implemented in the standard Python library NumPy (numpy.gradient).

## Supplementary Material



## Notes

Additional electrochemical data and Arrhenius analysis, electron microscopy images, photographs of the eQCM cell and experimental protocol, additional eQCM data, and discussion on the temperature dependence of the equilibrium potential (PDF)
