# Supplementary material for: The Interplay between Interfacial Solvation and Surface Kinetics Tunes the Selectivity between Hydrogen Evolution and Zinc Electrodeposition
Source: J Am Chem Soc. 2026 Jun 29;148(27):28377–89. doi: 10.1021/jacs.6c03962 (PMC13383738; doi:10.1021/jacs.6c03962)
Supplement: Supplementary file 1 [file ja6c03962_si_001.pdf]

Supporting Information

The interplay between interfacial solvation and surface kinetics tunes the selectivity between hydrogen evolution and zinc electrodeposition

Daniel Escalera-López, Raquel Anastacio, Carlos Gomez Rodellar, Wiebke Frandsen, Sebastian Z. Oener, Beatriz Roldan Cuenya**

Department of Interface Science, Fritz-Haber Institute of the Max Planck Society, Berlin 14195, Germany

*oener@fhi-berlin.mpg.de; roldan@fhi-berlin.mpg.de

Supplementary Note 1

The temperature can impact, both the open-circuit potential (thermodynamics) between the working and the reference electrode and, potentially, the overpotential-dependent surface concentrations (kinetics).

Thermodynamics at equilibrium: To understand the temperature dependence of the open-circuit potential, we need to consider the temperature dependence of the half reaction of the working and reference electrodes separately. The reduction potential, $E^0(T)$, is temperature dependent because of the temperature-dependent reaction thermodynamics and the Nernst equation for non-unit activities. Some temperature sensitivities can be found in Petersen et al.[1]

In general, for any temperature dependence of the equilibrium potential of a reduction half reaction (replacing activity for concentration)

$$E^0(T, 1\,M) = E^0(298\mathrm{K}, 1\,\mathrm{M}) + \frac{dE}{dT}(\mathrm{T} - 298K) \tag{1}$$

Additionally, for the concentration dependence of the reduction half-potential, we can write with the Nernst equation and the reaction quotient, Q,

$$E(T, Q) = E^0(T, 1\,M) - \frac{2.303RT}{nF}\log_{10} Q \tag{2}$$

The Nernst term on the right-hand side accounts for a temperature-dependent shift of the cell potential due to concentrations that deviate from the equilibrium concentration, i.e. when the reaction quotient, Q = [product]/[reactant] $\neq K_{\mathrm{eq}}$. Thus, the Nernst term is even important at a constant temperature, but with a changing reactant concentration at the interface.

Here, we consider two reactions, the HER in alkaline conditions ($2H_2O + 2e^- \rightarrow H_2 + 2OH^-$) with a temperature sensitivity of $dE^0(T)dT^{-1}$ of -8.35 mV/10˚C and the Zn electrodeposition ($Zn^{2+} + 2e^- \rightarrow Zn$) with $dE^0(T)dT^{-1}$ of -0.99 mV/10˚C.

We are using a reversible hydrogen electrode as reference electrode, i.e. the reaction and pH are identical to the working electrode when studying the reversible hydrogen electrode (RHE) on Zn. As a result, both reduction half potentials of the working and reference electrode shift equally with temperature and, thus, the temperature dependencies cancel. This would not be the case, if instead of the RHE, the standard hydrogen electrode (SHE, 1M H^+) would have been used, i.e. if a pH-gradient would exist inside the cell, or if a different reference reaction with a different or no pH-dependence would be used, such as at the Ag/AgCl electrode.

The Zn^{2+} reduction ($Zn^{2+} + 2e^- \rightarrow Zn$) has a low temperature dependence of $dE^0(T)dT^{-1}$ of -0.99 mV/10˚C, i.e. the right-hand side term in equation (1) provides -2.5 mV/25˚C. In equation 2, the product concentration in Q (the metallic Zn) can be set to 1M (unit activity for solids), and the reactant concentration (Zn^{2+}) is 1mM, but will only change slightly in this narrow temperature range. For n=2, the ($\frac{2.303RT}{nF}$) pre-factor in the Nernst term changes from 10˚C (28.1 mV) to 25˚C (29.6 mV) to 35˚C (30.5 mV). Given the 1mM ZnO concentration, $\log_{10} Q$ in the Nernst term provides a factor of three. In total, eq. 2 predicts a shift of 7.2 mV and eq. 1 a shift of -2.5 mV over the 25˚C temperature window, between 10˚C and 35˚C, resulting in a total theoretical shift of ~ 5 mV in total. To test this, we performed additional experiments, as shown in Suppl. Fig. 21 and find an open-circuit potential shift of 2

mV/10˚C, i.e. 5 mV in total, in good agreement with the theoretical value. In Supplementary Figure 25, we show the impact of different temperature-dependent shifts for an experimental data set of the main, confirming the negligible impact of a 2 mV/10˚C shift.

At applied overpotential: In general, overpotential-dependent changes in the reactant or product concentrations could lead to substantial changes of the open-circuit/cell potential. However, in the main, we already discuss in detail that related mass transport effects are likely absent at the low overpotential range studied here.

Temperature-dependent concentration changes are likely absent in our data, as is indicated by the high Arrhenius linear regression values at different potential. Any substantial temperature-dependent concentration change would lead to non-linear Arrhenius curves. The same holds for intermediate coverages, e.g. Zn-H. Note, all intermediate coverages are necessarily temperature-dependent. However, the temperature window where these changes can be observed or are important varies drastically between different reactants/intermediates.

Finally, whereas we neglect temperature- or overpotential-dependent changes of the Zn^{2+}/Zn or H_2O/H_2 concentrations for the narrow temperature range studied, we cannot exclude overpotential-dependent intermediate coverages on the catalyst surface. Such effects are likely less important for metal deposition, but for the HER, we and others recently detailed that overpotential-dependent coverages can still lead to linear Arrhenius curves, due to an underlying multi-step sequence and overpotential-dependent rate limiting steps[2–4]. However, as for the reactant and product concentrations, a strong temperature-dependent intermediate coverage would quickly lead to non-linear Arrhenius, too.

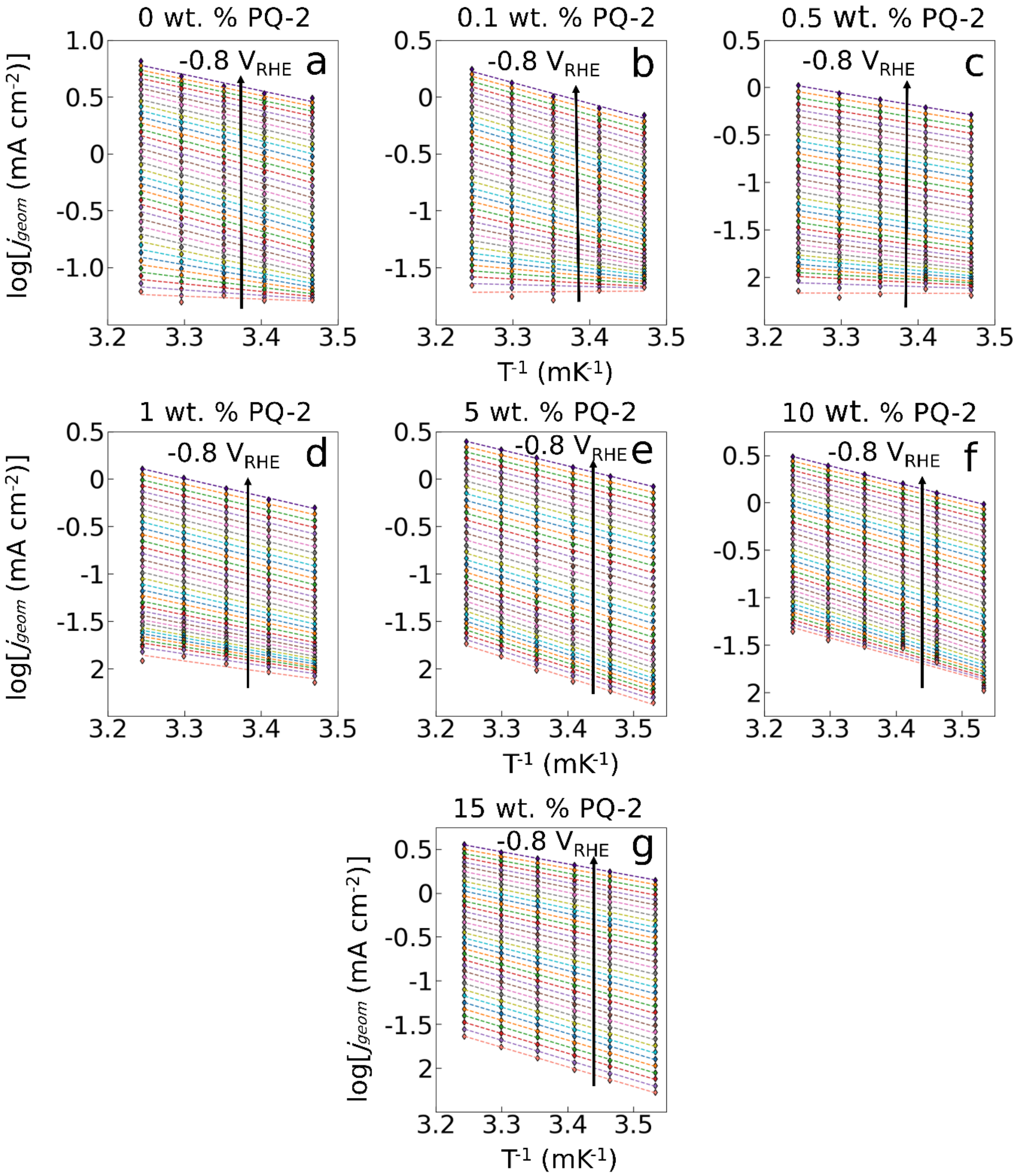

Supplementary Figure 1. a-g) Arrhenius analysis of multi-step chronoamperometric measurements presented in Figure 1 and Supplementary Figure S3, obtained in 0.1 M KOH electrolytes (pH 13) with respect to polyquaternium-2 (PQ-2) content (0 to 15 wt.%). Linear regression R^2 values are well above 0.9 (≥ 0.95) with the exception of some of the lowest overpotentials and currents. The presented data were not corrected for equilibrium potential temperature dependence given its almost negligible shift across the operating temperature range (-0.99 mV 10 °C^{-1}).

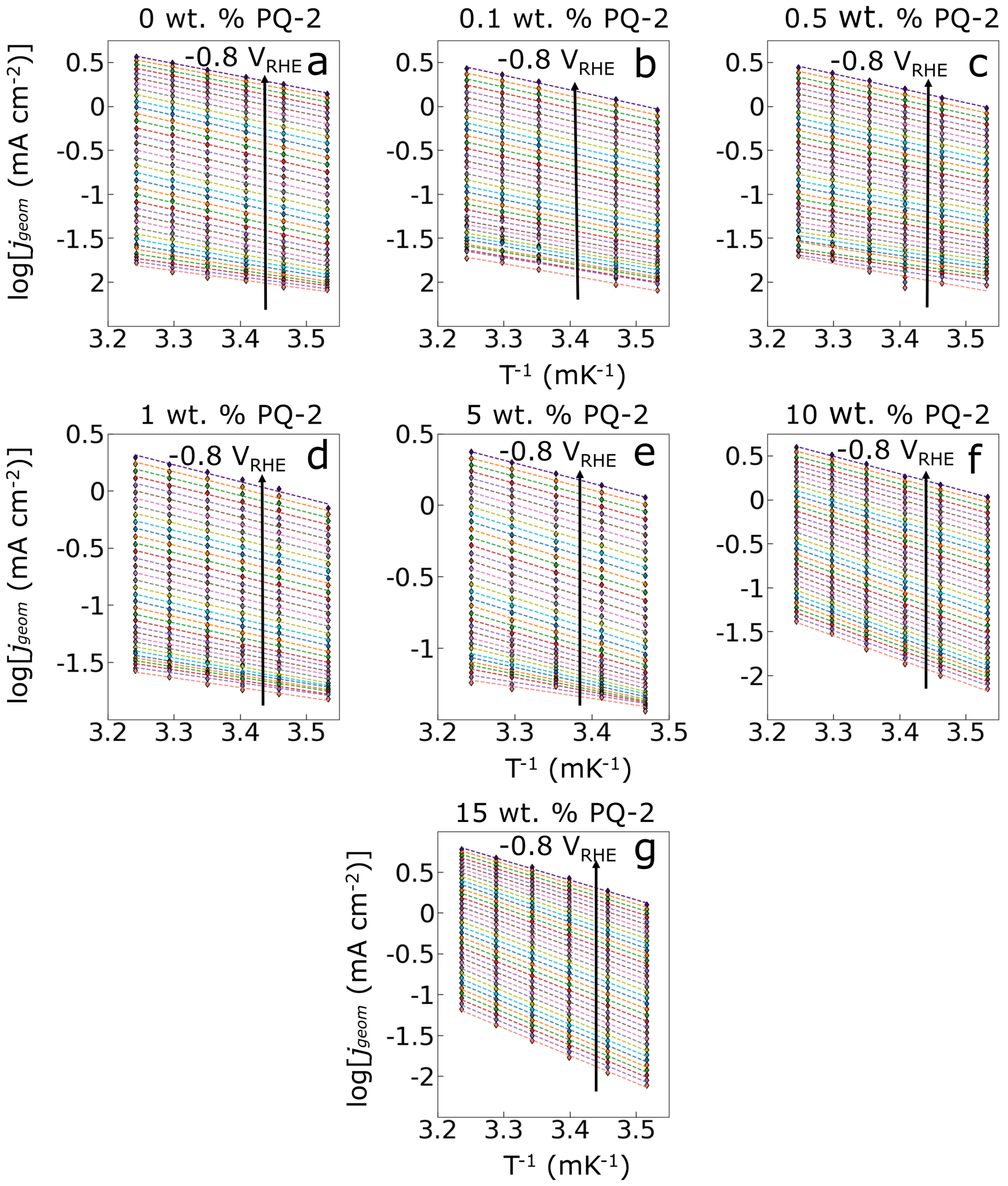

Supplementary Figure 2. a-g) Arrhenius analysis of multi-step chronoamperometric measurements presented in Figure 2 and Supplementary Figure S4-5, obtained in 0.1 M KOH electrolytes (pH 13) in presence of dissolved zincate (1mM ZnO) with respect to polyquaternium-2 (PQ-2) content (0 to 15 wt.%). Linear regression R^2 values are well above 0.9 (≥ 0.95). The data were not corrected for equilibrium potential temperature dependence given its almost negligible shift across the operating temperature range (-0.99 mV 10 °C^{-1}).

a 0 wt. % PQ-2

η (V)	R^2
-0.45	0.337
-0.5	0.908
-0.55	0.945
-0.60	0.951
-0.65	0.955
-0.70	0.946
-0.75	0.941
-0.80	0.943

b 0.1 wt. % PQ-2

η (V)	R^2
-0.45	0.650
-0.5	0.672
-0.55	0.879
-0.60	0.948
-0.65	0.969
-0.70	0.974
-0.75	0.977
-0.80	0.981

c 0.5 wt. % PQ-2

η (V)	R^2
-0.45	0.227
-0.5	0.883
-0.55	0.978
-0.60	0.995
-0.65	0.997
-0.70	0.997
-0.75	0.999
-0.80	0.999

d 1 wt. % PQ-2

η (V)	R^2
-0.45	0.825
-0.5	0.955
-0.55	0.990
-0.60	0.997
-0.65	0.999
-0.70	0.999
-0.75	0.998
-0.80	0.995

e 5 wt. % PQ-2

η (V)	R^2
-0.45	0.991
-0.5	0.990
-0.55	0.990
-0.60	0.991
-0.65	0.994
-0.70	0.997
-0.75	0.999
-0.80	0.999

f 10 wt. % PQ-2

η (V)	R^2
-0.45	0.887
-0.5	0.955
-0.55	0.987
-0.60	0.998
-0.65	0.999
-0.70	0.999
-0.75	0.999
-0.80	0.999

g 15 wt. % PQ-2

η (V)	R^2
-0.45	0.999
-0.5	0.999
-0.55	0.997
-0.60	0.997
-0.65	0.998
-0.70	0.992
-0.75	0.996
-0.80	0.999

Supplementary Figure 3. a-g) Compiled heatmap for R^2 values extracted from Arrhenius analysis of multi-step chronoamperometric measurements presented in Figure 1 and Supplementary Figure S5, obtained in 0.1 M KOH electrolytes (pH 13) with respect to polyquaternium-2 (PQ-2) content (0 to 15 wt.%). Colour coding for heatmaps: red ($R^2 \leq 0.85$), orange ($R^2 \leq 0.95$), green ($R^2 \leq 0.99$), turquoise ($R^2 > 0.99$). Linear regression R^2 values are generally well above 0.9 (≥ 0.95), except for some low overpotentials and currents.

a 0 wt. % PQ-2

η (V)	R^2
-0.45	0.957
-0.5	0.989
-0.55	0.994
-0.60	0.998
-0.65	0.998
-0.70	0.999
-0.75	0.999
-0.80	0.998

b 0.1 wt. % PQ-2

η (V)	R^2
-0.45	0.995
-0.5	0.968
-0.55	0.997
-0.60	0.998
-0.65	0.999
-0.70	0.999
-0.75	0.998
-0.80	0.996

c 0.5 wt. % PQ-2

η (V)	R^2
-0.45	0.809
-0.5	0.868
-0.55	0.936
-0.60	0.968
-0.65	0.983
-0.70	0.993
-0.75	0.998
-0.80	0.996

d 1 wt. % PQ-2

η (V)	R^2
-0.45	0.982
-0.5	0.979
-0.55	0.999
-0.60	0.998
-0.65	0.997
-0.70	0.994
-0.75	0.990
-0.80	0.978

e 5 wt. % PQ-2

η (V)	R^2
-0.45	0.837
-0.5	0.956
-0.55	0.985
-0.60	0.995
-0.65	0.998
-0.70	0.999
-0.75	0.998
-0.80	0.999

f 10 wt. % PQ-2

η (V)	R^2
-0.45	0.993
-0.5	0.994
-0.55	0.995
-0.60	0.998
-0.65	0.998
-0.70	0.998
-0.75	0.997
-0.80	0.997

g 15 wt. % PQ-2

η (V)	R^2
-0.45	0.997
-0.5	0.998
-0.55	0.999
-0.60	0.999
-0.65	0.998
-0.70	0.996
-0.75	0.995
-0.80	0.994

Supplementary Figure 4. a-g) Compiled heatmap for R^2 values extracted from Arrhenius analysis of multi-step chronoamperometric measurements presented in Figure 2 and Suppl. Figs. S6-7, obtained in 0.1 M KOH electrolytes (pH 13) in presence of dissolved zincate (1mM ZnO) with respect to polyquaternium-2 (PQ-2) content (0 to 15 wt.%). Colour coding for heatmaps: red ($R^2 \leq 0.85$), orange ($R^2 \leq 0.95$), green ($R^2 \leq 0.99$), turquoise ($R^2 > 0.99$). Linear regression R^2 values are generally well above 0.9 (≥ 0.95), except for some low overpotentials and currents.

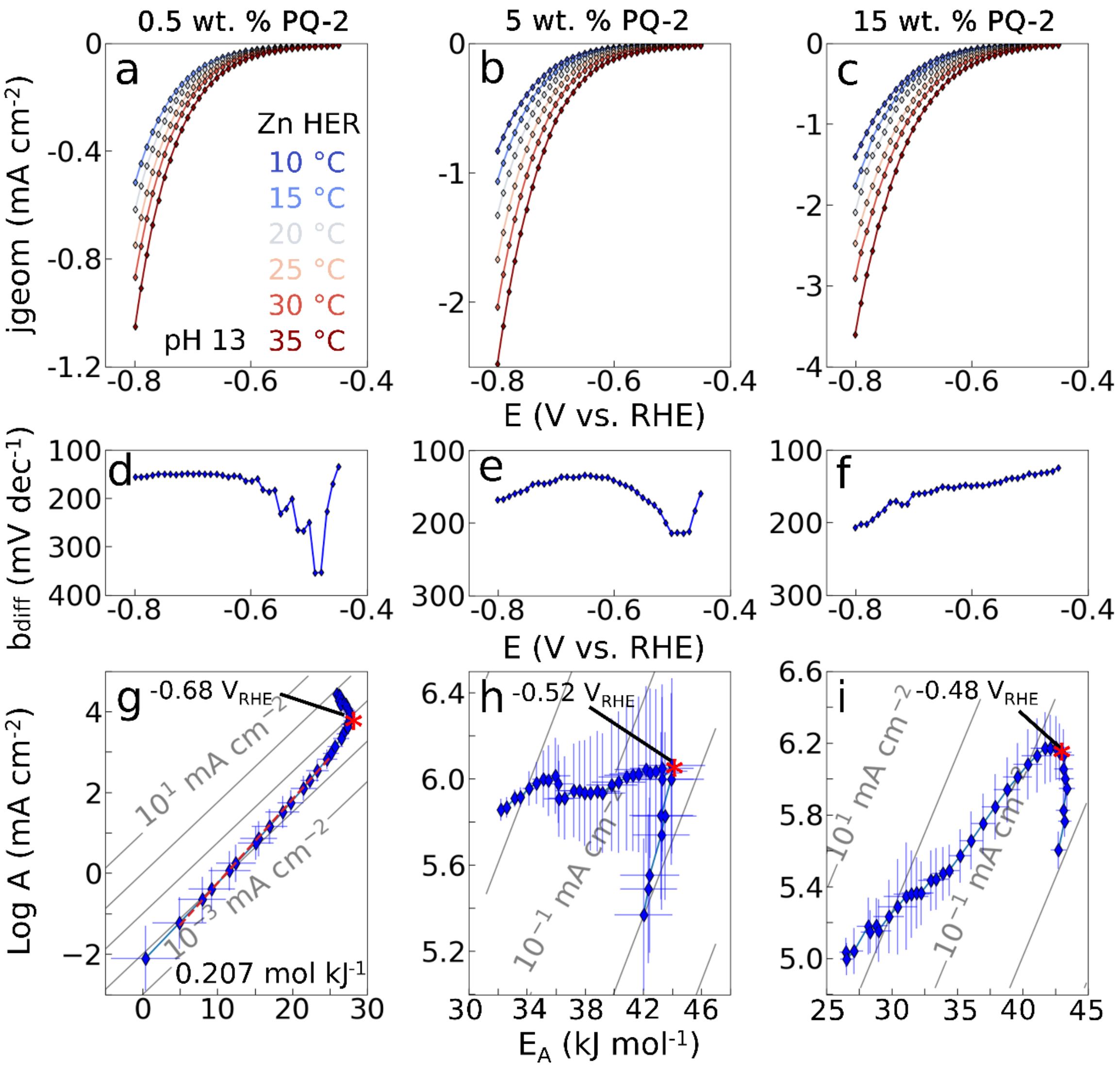

Supplementary Figure 5. a-c) Representative current-averaged multi-step chronoamperometric measurements at different temperatures (10-35 °C), d-f) differential Tafel slope analysis, and g-i) bias-dependent compensation plots [log A(η) vs. E_A (η)] obtained from Arrhenius analysis of multi-step chronoamperometric measurements presented obtained in 0.1 M KOH electrolytes (pH 13) with respect to polyquaternium-2 (PQ-2) content (0.5, 5 and 15 wt.%). Turning potentials, marked in panel g-i as the potential with the highest E_A (red asterisk), denote the transition from kinetics presumably dominated by interfacial (de)solvation processes to kinetics governed by the energetics of surface intermediates.

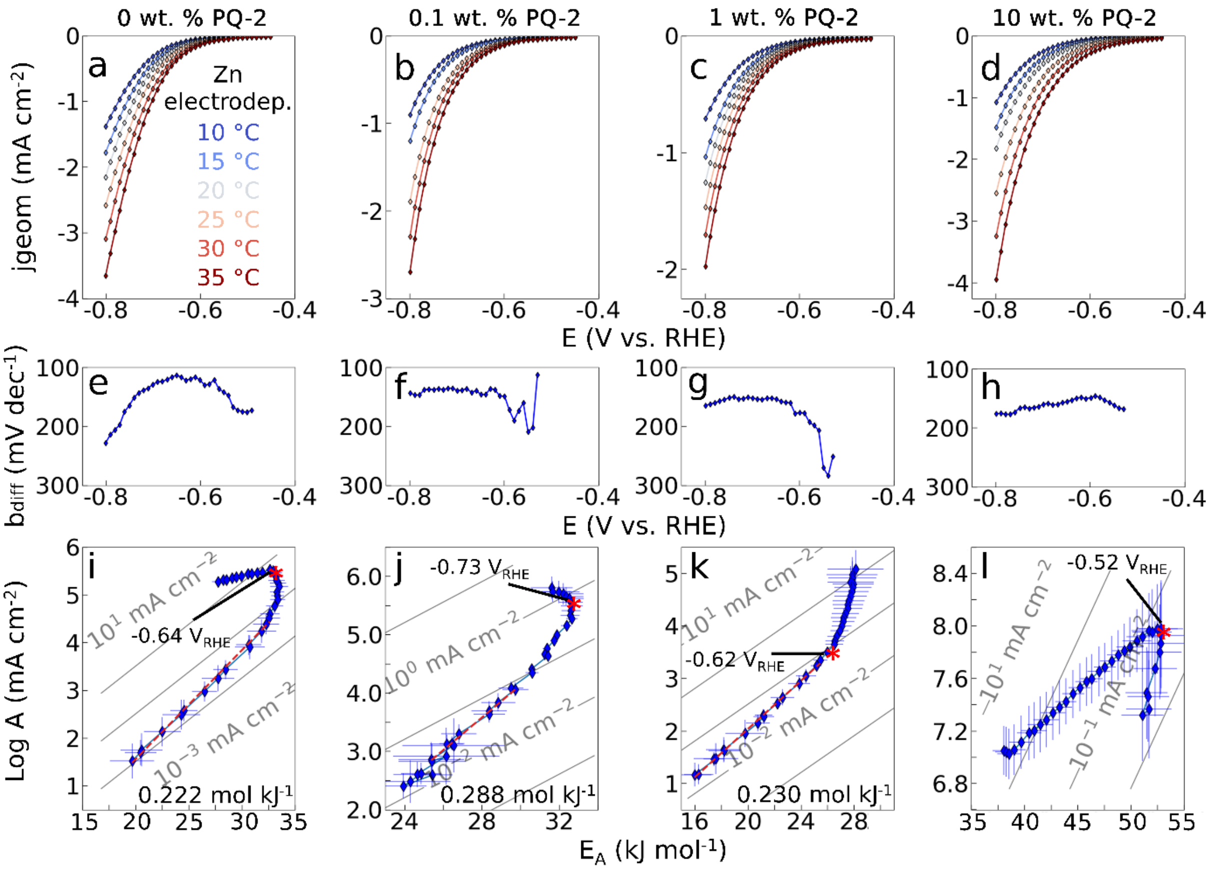

Supplementary Figure 6. a-c) Representative current-averaged multi-step chronoamperometric measurements at different temperatures (10-35 °C), e-h) differential Tafel slope analysis, and i-l) bias-dependent compensation plots [log $A(\eta)$ vs. $E_A(\eta)$] obtained from Arrhenius analysis of multi-step chronoamperometric measurements presented obtained in 0.1 M KOH electrolytes (pH 13) in presence of dissolved zincate (1mM ZnO) with respect to polyquaternium-2 (PQ-2) content (0, 0.1, 1 and 10 wt.%). Turning potentials, marked in S4i-l as the potential with the highest E_A (red asterisk), denote the transition from kinetics presumably dominated by interfacial (de)solvation processes to kinetics governed by the energetics of surface intermediates, as described by the traditional Butler-Volmer equation with an approximately constant pre-factor.

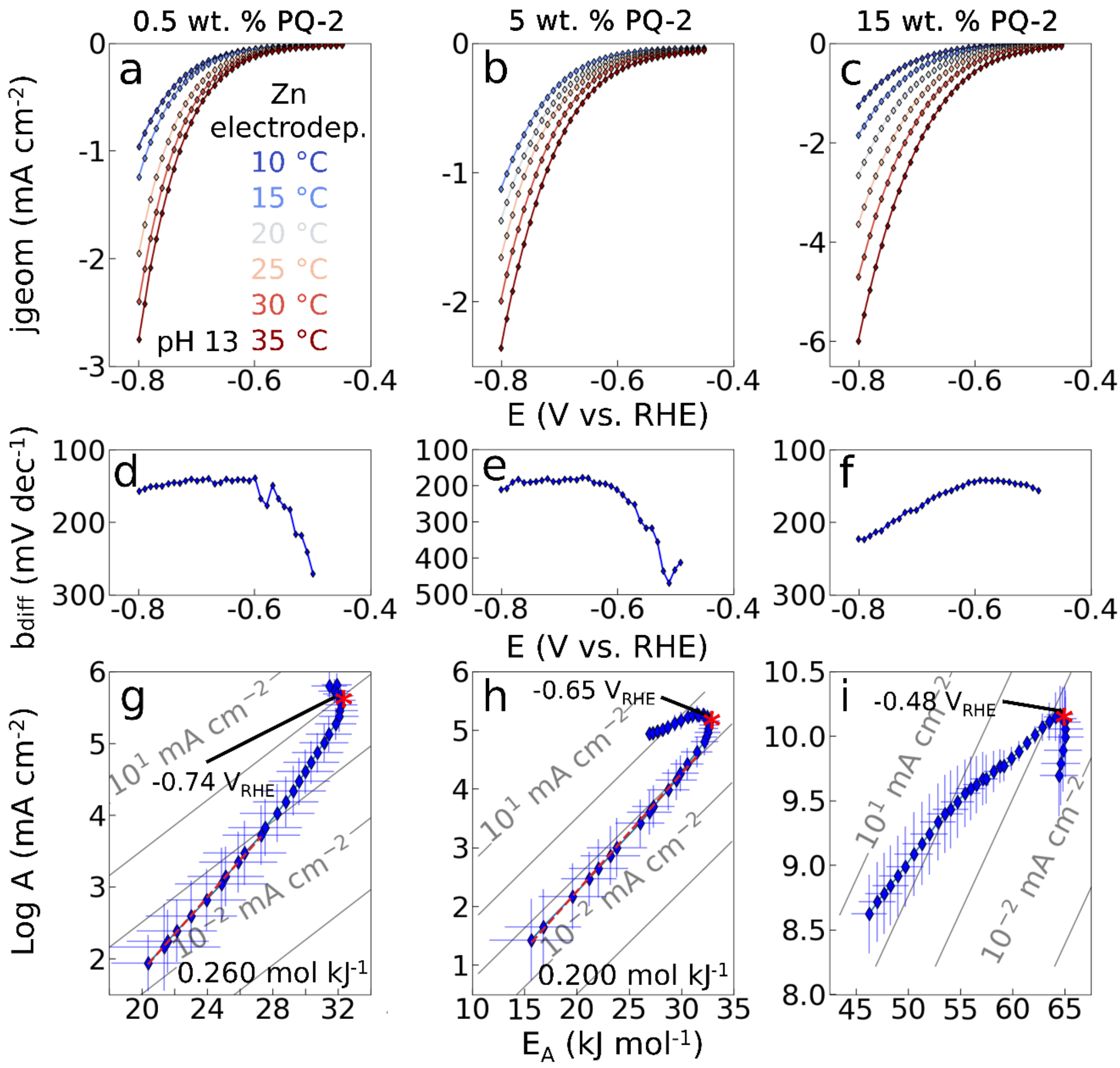

Supplementary Figure 7. a-c) Representative current-averaged multi-step chronoamperometric measurements at different temperatures (10-35 °C), d-f) differential Tafel slope analysis, and g-i) bias-dependent compensation plots [log $A(\eta)$ vs. $E_A(\eta)$] obtained from Arrhenius analysis of multi-step chronoamperometric measurements presented obtained in 0.1 M KOH electrolytes (pH 13) in presence of dissolved zincate (1mM ZnO) with respect to polyquaternium-2 (PQ-2) content (0.5, 5 and 15 wt.%). Turning potentials, marked in S4g-i as the potential with the highest E_A (red asterisk), denote the transition from kinetics presumably dominated by interfacial (de)solvation processes to kinetics governed by the energetics of surface intermediates, as described by the traditional Butler-Volmer equation with an approximately constant pre-factor.

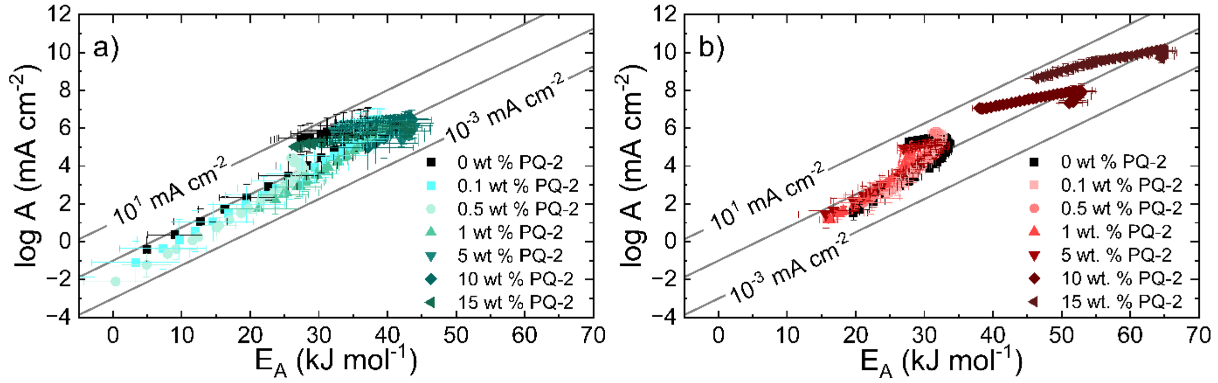

Supplementary Figure 8. Overlayed compensation plots [log $A(\eta)$ vs. $E_A\,(\eta)$] in absence (a) or presence of dissolved zincate (b) with polyquaternium-2 (PQ-2) content (0-15 wt.%).

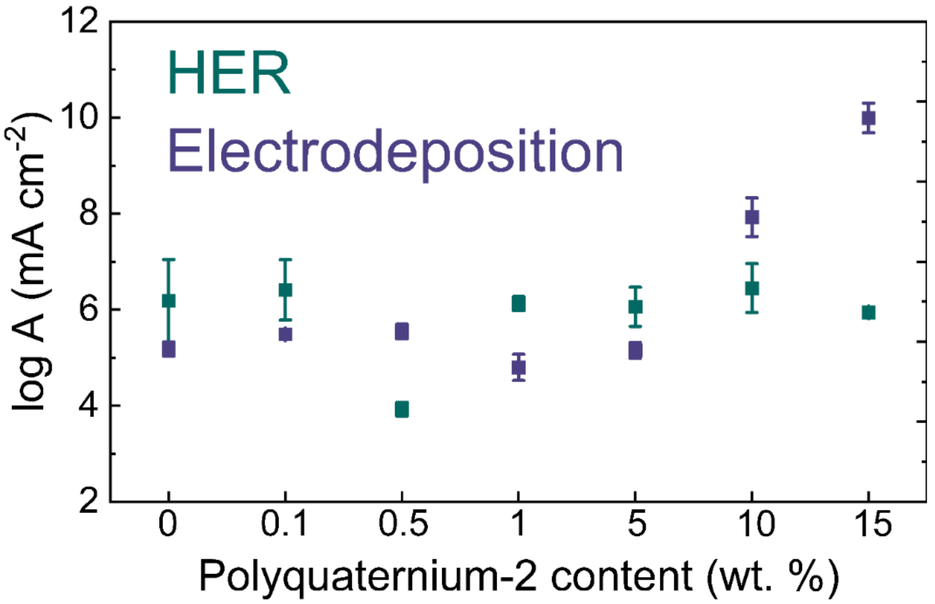

Supplementary Figure 9. Pre-exponential factor [log $A(\eta)$] linked to maximum [$E_A(\eta)$] values from compensation plots in Figures 1-2 and S1-2 in absence (green) or in the presence of dissolved zincate (1mM ZnO, purple) with respect to polyquaternium-2 (PQ-2) content (0 to 15 wt.%).

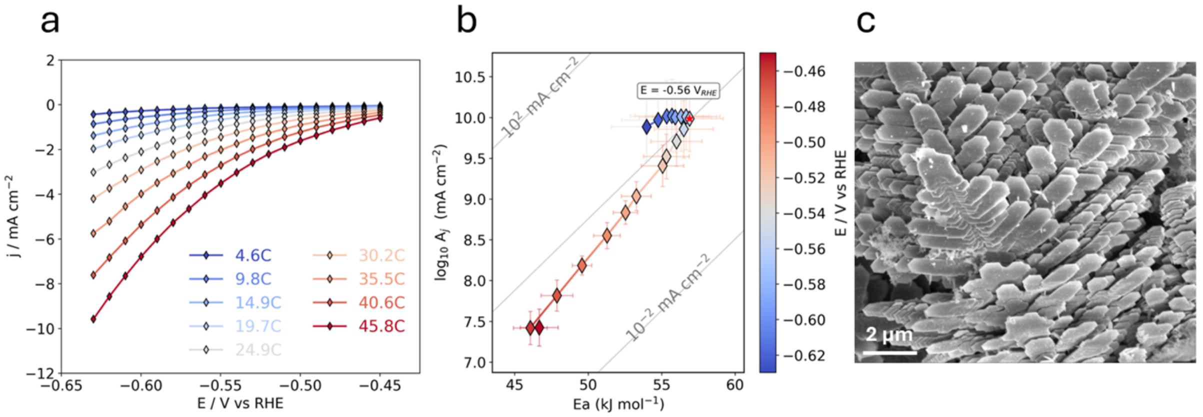

Supplementary Figure 10. Polymer-free high-salt control of Zn electrodeposition in 0.1 M KOH + 1 mM ZnO + 1 *m* $NaClO_4$. (a) Temperature-dependent polarization curves extracted from multistep chronoamperometry between 4.6 and 45.8 °C. (b) Arrhenius-derived kinetic map ($\log_{10}A$ vs. E_a), with each point color-coded by the applied potential (vs. RHE). Similar to the 10 wt.% PQ-2, the 1 *m* $NaClO_4$ electrolyte induces an absolute shift to much higher pre-factors and apparent activation energies compared to the case without $NaClO_4$ (e.g., compare to Fig. 2e) and a potential-dependent turnover around −0.56 V_{RHE}. Overall, the impact of 1 *m* $NaClO_4$ is very similar to the case 10 wt. % PQ-2 (compare to Fig. 2h) in terms of the absolute activation parameters. However, compared to the PQ-2, the absolute current densities are substantially higher (the apparent activation parameter curve is shifted toward higher isocurrent lines). This also induces an impact of mass transport in the polarization curves at lower negative potentials. (c) Representative SEM image of the resulting Zn deposit after the multi-step chronoamperometry measurements up to -0.8 V_{RHE}, showing plate-like crystallites assembled into rough, branched dendritic structures.

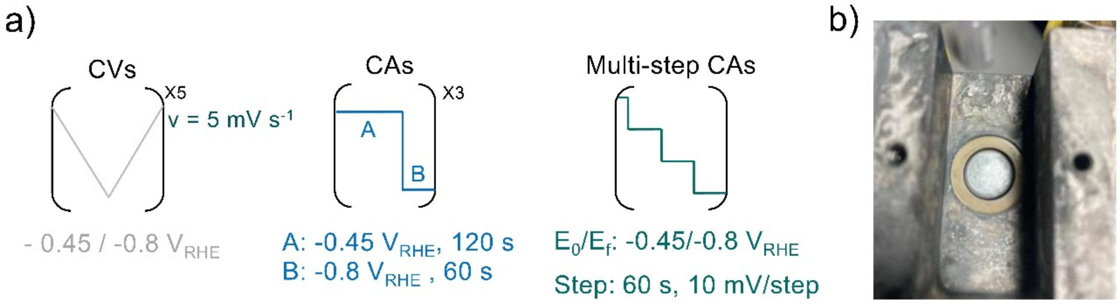

Supplementary Figure 11. a) Electrochemical protocols employed during eQCM measurements. b) representative Zn thin film obtained after pulsed deposition on a Au-coated quartz crystal. Electrolytic bath: 0.7 M ZnO, 30 % KOH.

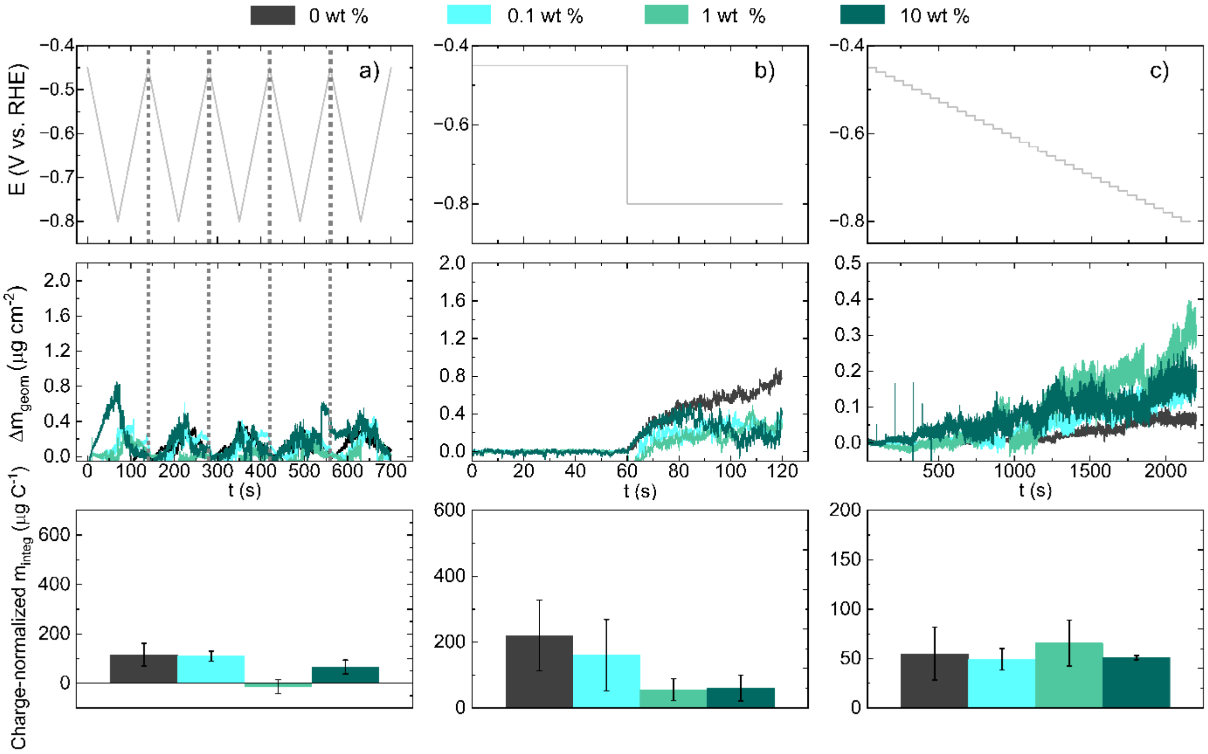

Supplementary Figure 12. Time-dependent mass gain profiles obtained in **zincate-free** KOH electrolyte (pH 13) with respect to PQ-2 contents during a) sequential cyclic voltammetry acquisition, b) on/off chronoamperometric holds and c) multi-step chronoamperometric holds on Zn-plated Au eQCM electrodes. Bottom panels: charge-normalized mass gain (m_{integ}) stemming from the voltammograms/chronoamperograms shown in panels a-c (middle panels).

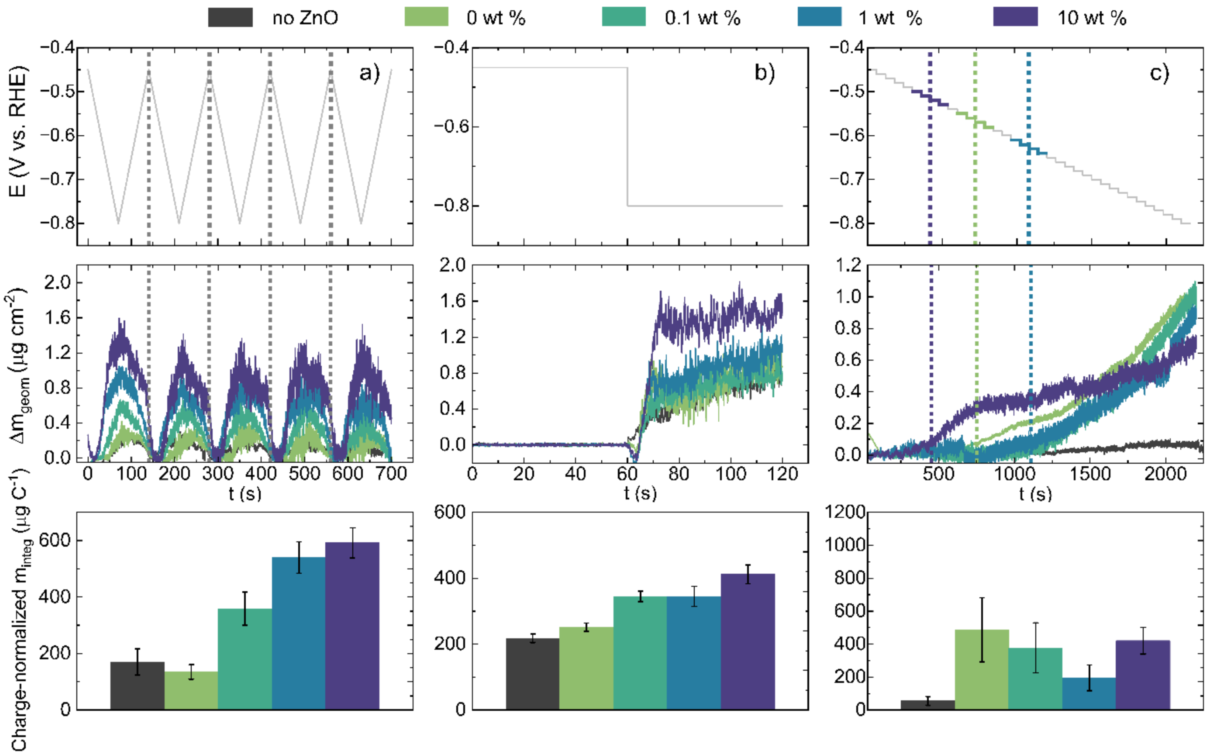

Supplementary Figure 13. Time-dependent mass gain profiles obtained in the absence and in the presence of **zincate** (1mM ZnO) with respect to PQ-2 contents in the KOH electrolyte (pH 13) during a) sequential cyclic voltammetry acquisition, b) on/off chronoamperometric holds and c) multi-step chronoamperometric holds on Zn-plated Au eQCM electrodes. Bottom panels: charge-normalized mass gain (m_{integ}) stemming from the voltammograms/chronoamperograms shown in panels a-c (middle panels).

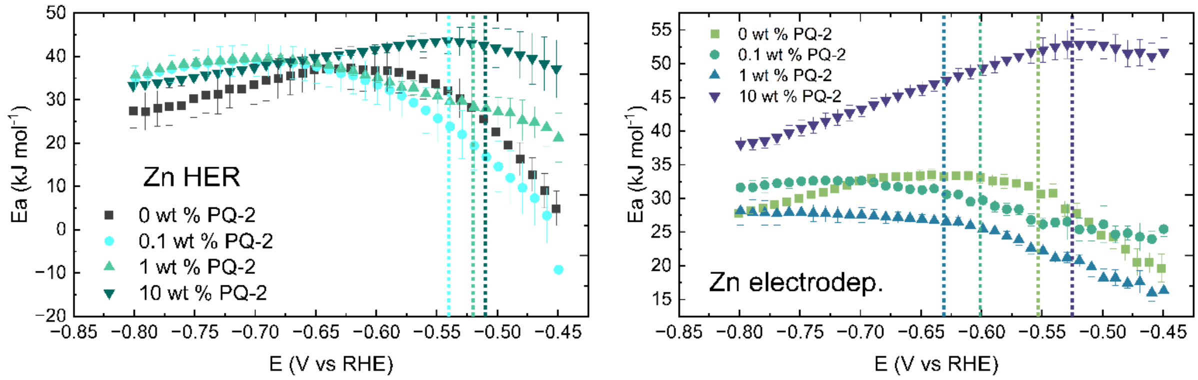

Supplementary Figure 14. Activation energies [E_A (η)] extracted from compensation plots in Figure 3 shown as a function of the potential bias in absence (left) or in presence of dissolved zincate (right) with respect to polyquaternium-2 (PQ-2) content (0, 0.1, 1 and 10 wt.%). Dashed lines represent mass gain onsets extracted from multi-step chronoamperometric eQCM measurements. For pristine and Zn-containing electrolytes, mass gains are ascribed to PQ-2 adsorption and Zn electrodeposition, respectively.

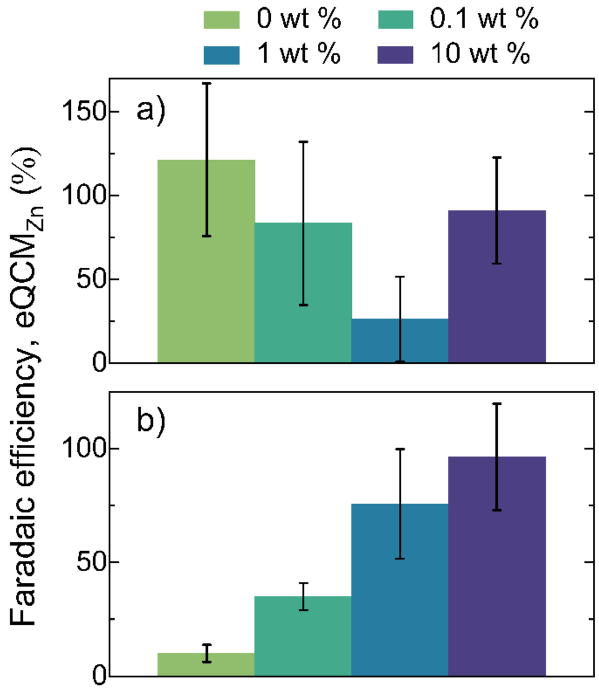

Supplementary Figure 15. Zn plating faradaic efficiencies (FEs) during a) multi-step and b) on-off chronoamperometric holds with respect to polyquaternium-2 (PQ-2) content (0, 0.1, 1 and 10 wt.%).

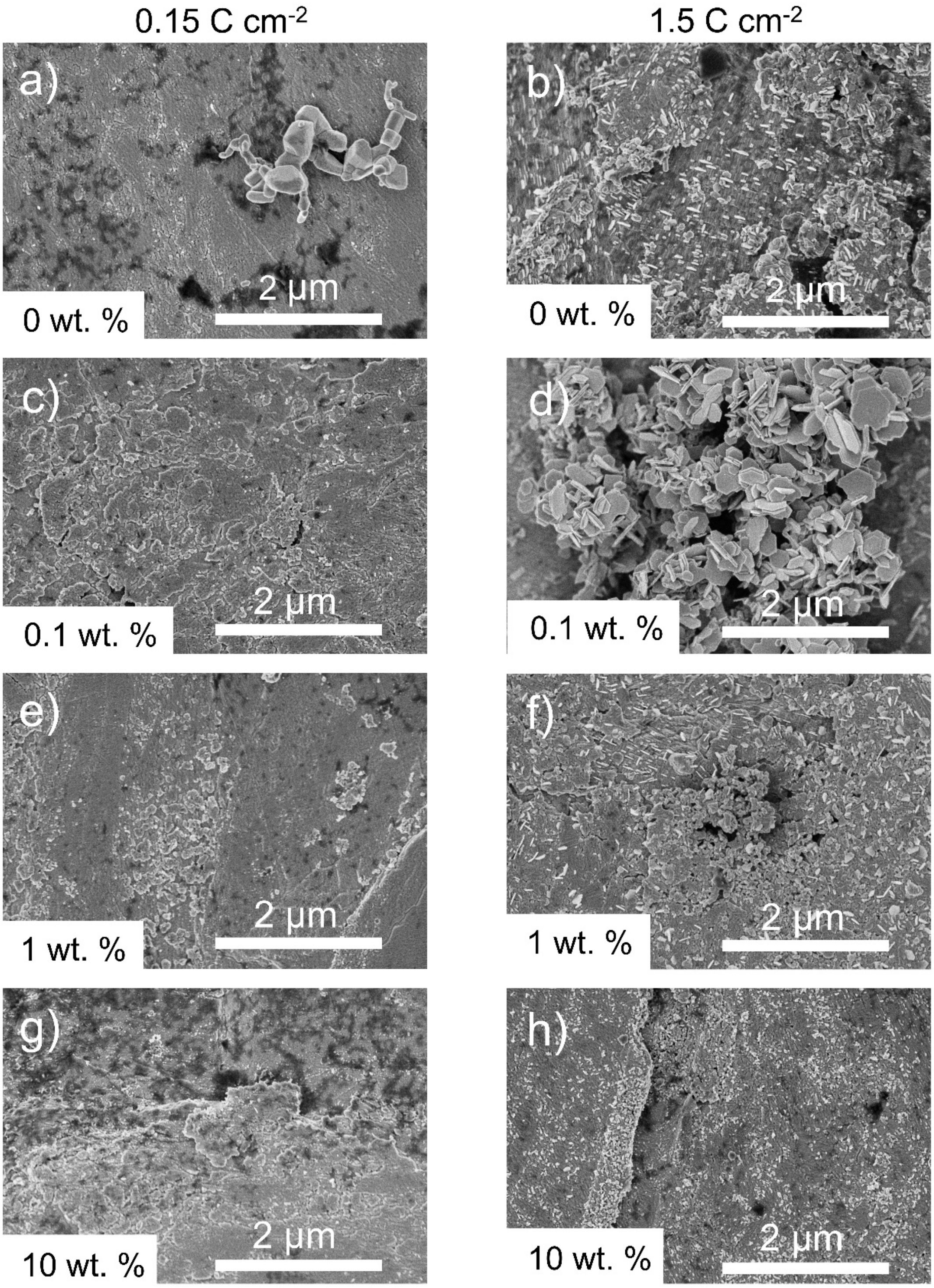

Supplementary Figure 16. SEM micrographs obtained for Zn film electrodeposits at charge density cut-off values $<\sigma_q>$ = -0.15 and -1.5 C cm^{-2}. Electrodeposits were prepared in 0.1 M KOH electrolytes (pH 13) with different polyquaternium-2 (PQ-2) contents: a-b) 0 wt. %, c-d) 0.1 wt. %, e-f) 1 wt. %, and g-h) 10 wt. %. SEM micrograph magnification: 25000X.

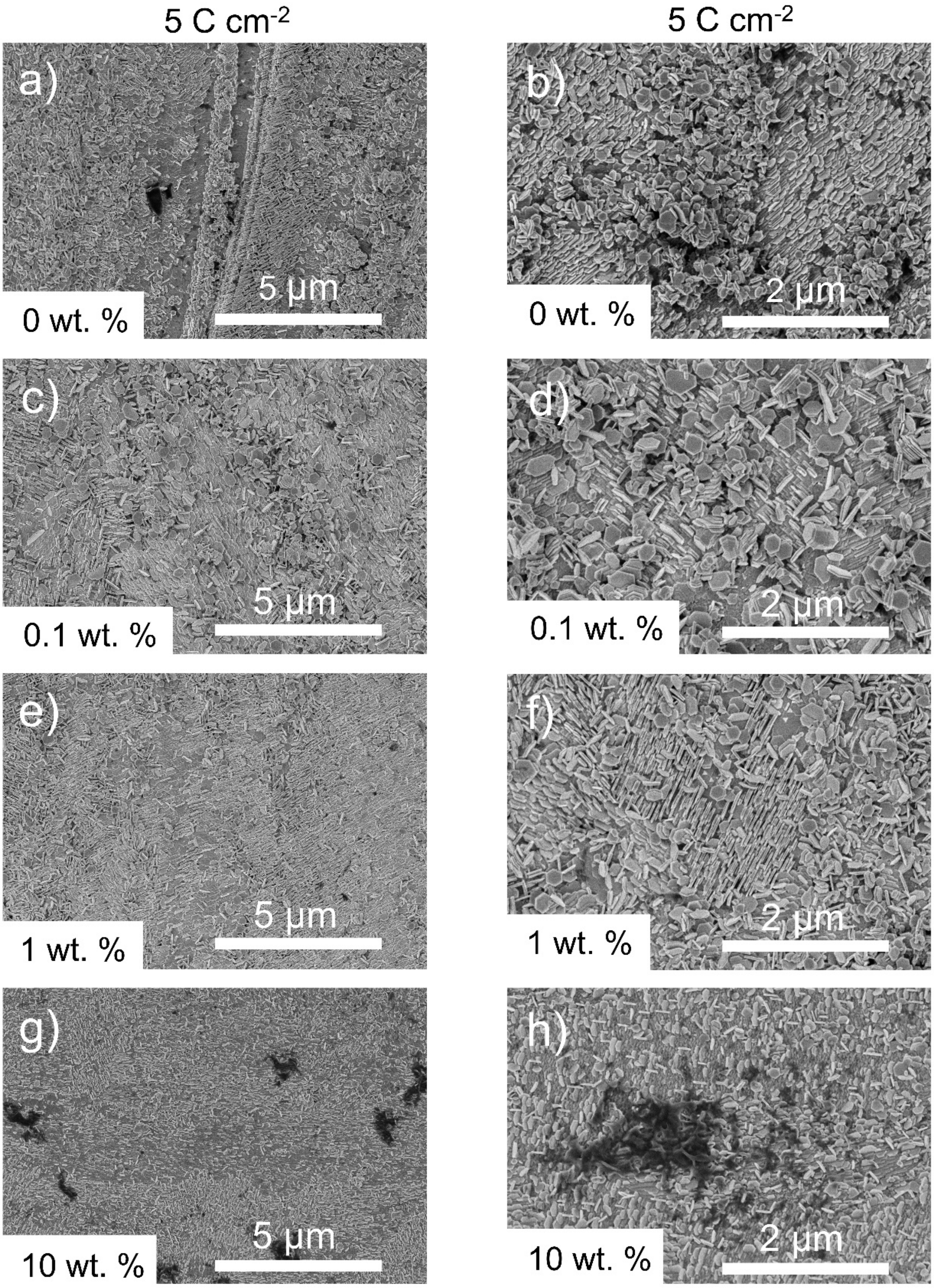

Supplementary Figure 17. SEM micrographs obtained for Zn film electrodeposits at charge density cut-off value <σ_q> = -5 C cm^{-2}. Electrodeposits were prepared in 0.1 M KOH electrolytes (pH 13) with different polyquaternium-2 (PQ-2) contents: a-b) 0 wt. %, c-d) 0.1 wt. %, e-f) 1 wt. %, and g-h) 10 wt. %. SEM micrograph magnification: 10000X (left column), 25000X (right column).

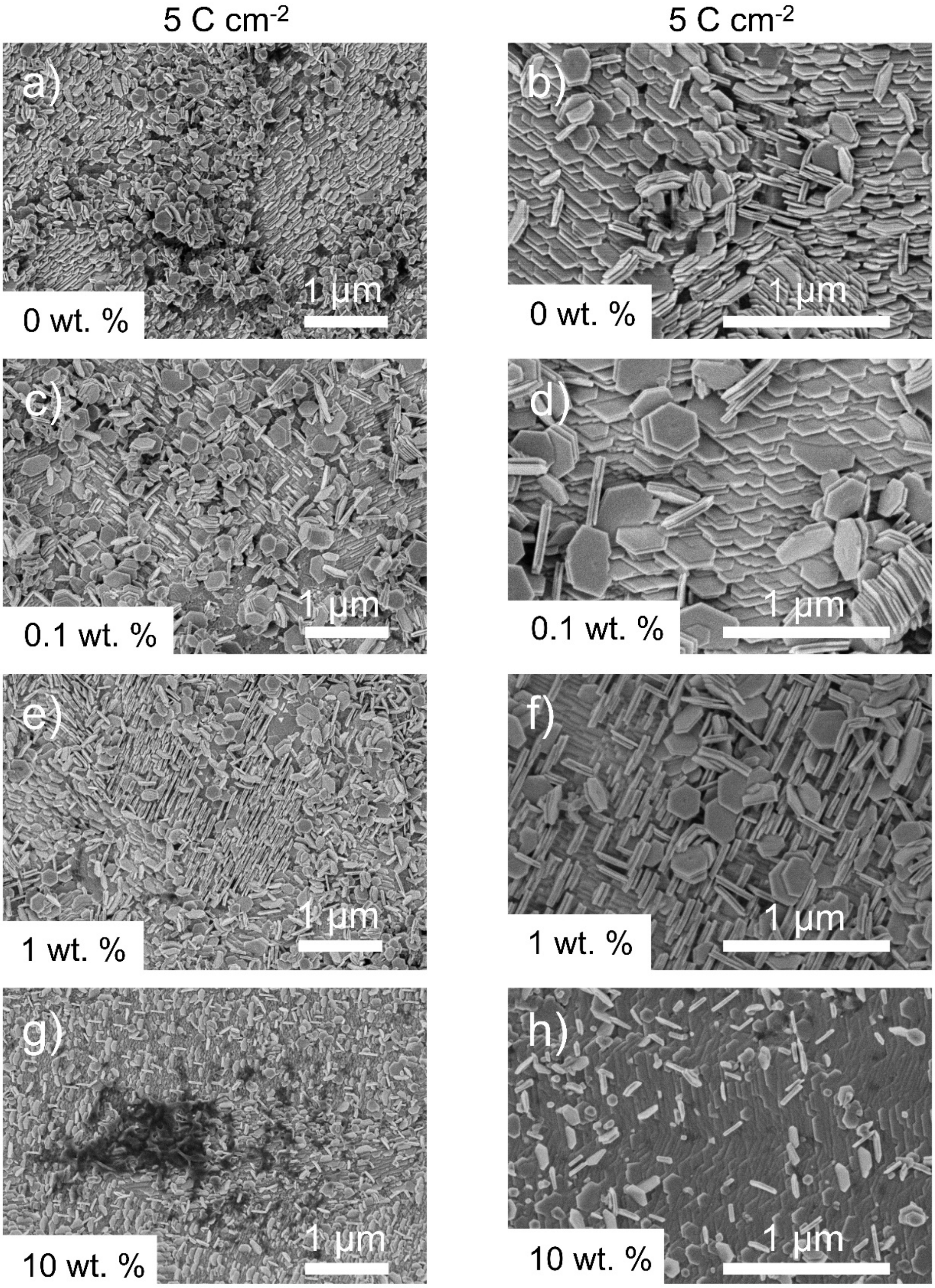

Supplementary Figure 18. SEM micrographs obtained for Zn film electrodeposits at charge density cut-off value $<\sigma_q>$ = -5 C cm^{-2}. Electrodeposits were prepared in 0.1 M KOH electrolytes (pH 13) with different polyquaternium-2 (PQ-2) contents: a-b) 0 wt. %, c-d) 0.1 wt. %, e-f) 1 wt. %, and g-h) 10 wt. %. SEM micrograph magnification: 25000X (left column), 50000X (right column).

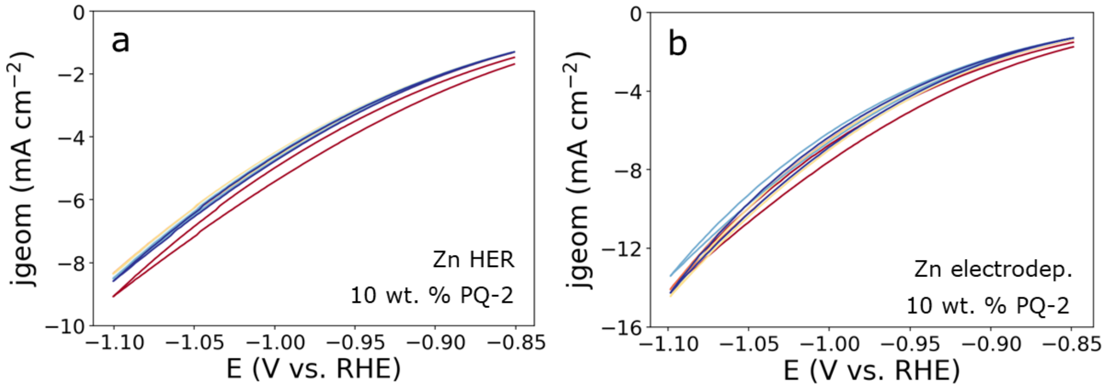

Supplementary Figure 19. Representative cyclic voltammograms obtained during Zn electrode preconditioning (-0.85 to -1.1 V_{RHE}, 100 cycles, 100 mVs^{-1}) for a) 0.1 M KOH and b) 0.1 M KOH in presence of dissolved zincate (1mM ZnO), both containing 10 wt. % polyquaternium-2. Labels: 1st cycle (red), 25th cycle (orange), 50th cycle (yellow), 75th cycle (light blue), 100th cycle (dark blue).

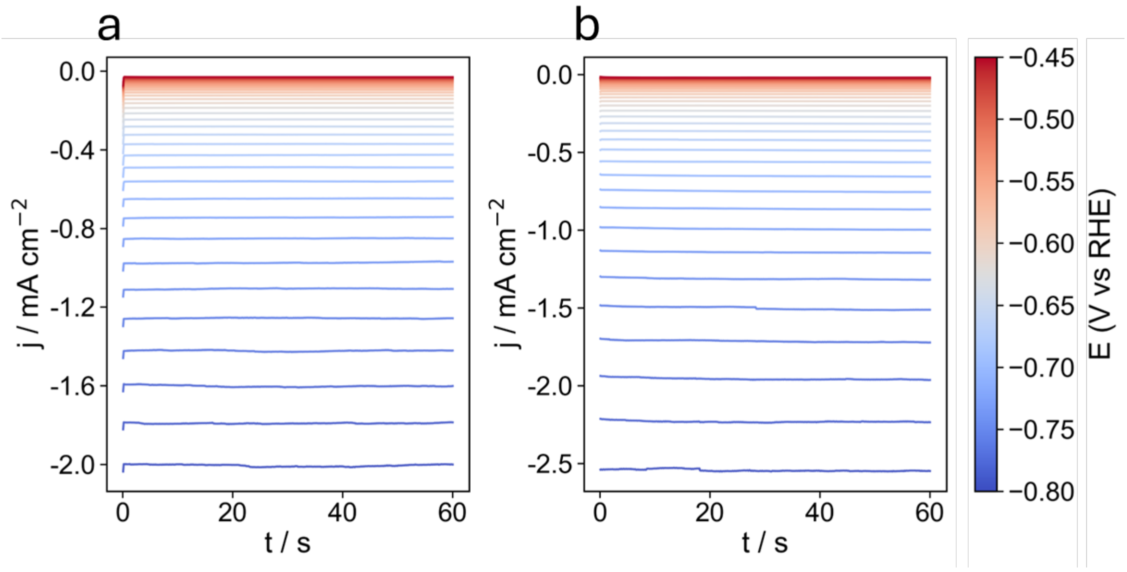

Supplementary Figure 20. Representative chronoamperometric current–time transients recorded after Zn-foil preconditioning by cyclic voltammetry (−0.85 to −1.10 V vs RHE, 100 cycles, 100 mV s^{-1}) for a) 0.1 M KOH + 10 wt.% PQ-2 and b) 0.1 M KOH + 1 mM ZnO + 10 wt.% PQ-2. Each trace corresponds to a 60 s potential hold in the subsequent multistep chronoamperometric protocol, with the applied potential indicated by the color scale.

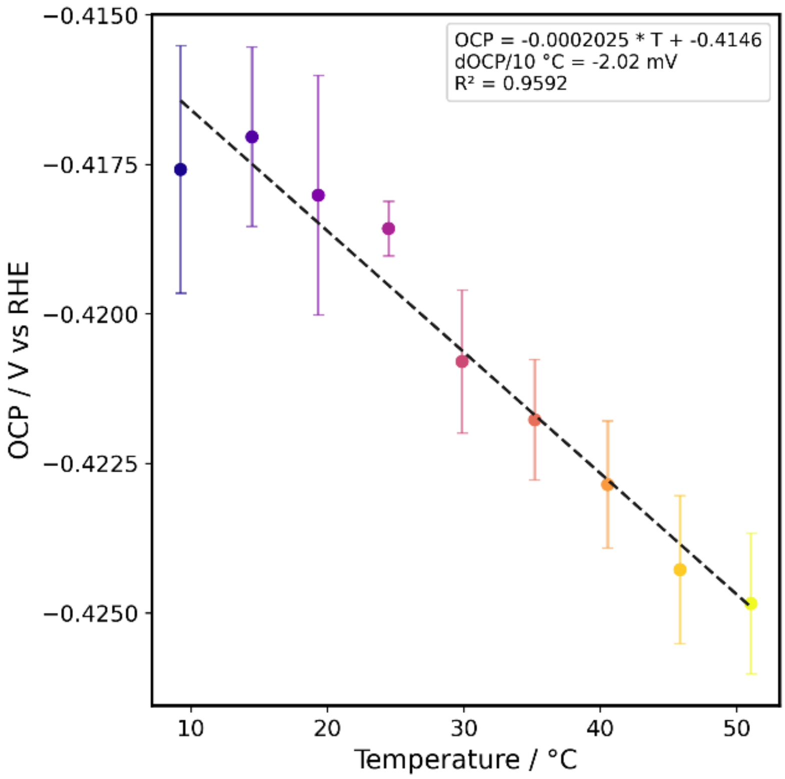

Supplementary Figure 21. Temperature dependence of the open-circuit potential (OCP) measured in 0.1 M KOH + 1 mM ZnO. The OCP shifts linearly with temperature by −2.02 mV per 10 °C, showing that the equilibrium-potential drift over the experimental temperature window is small and negligible.

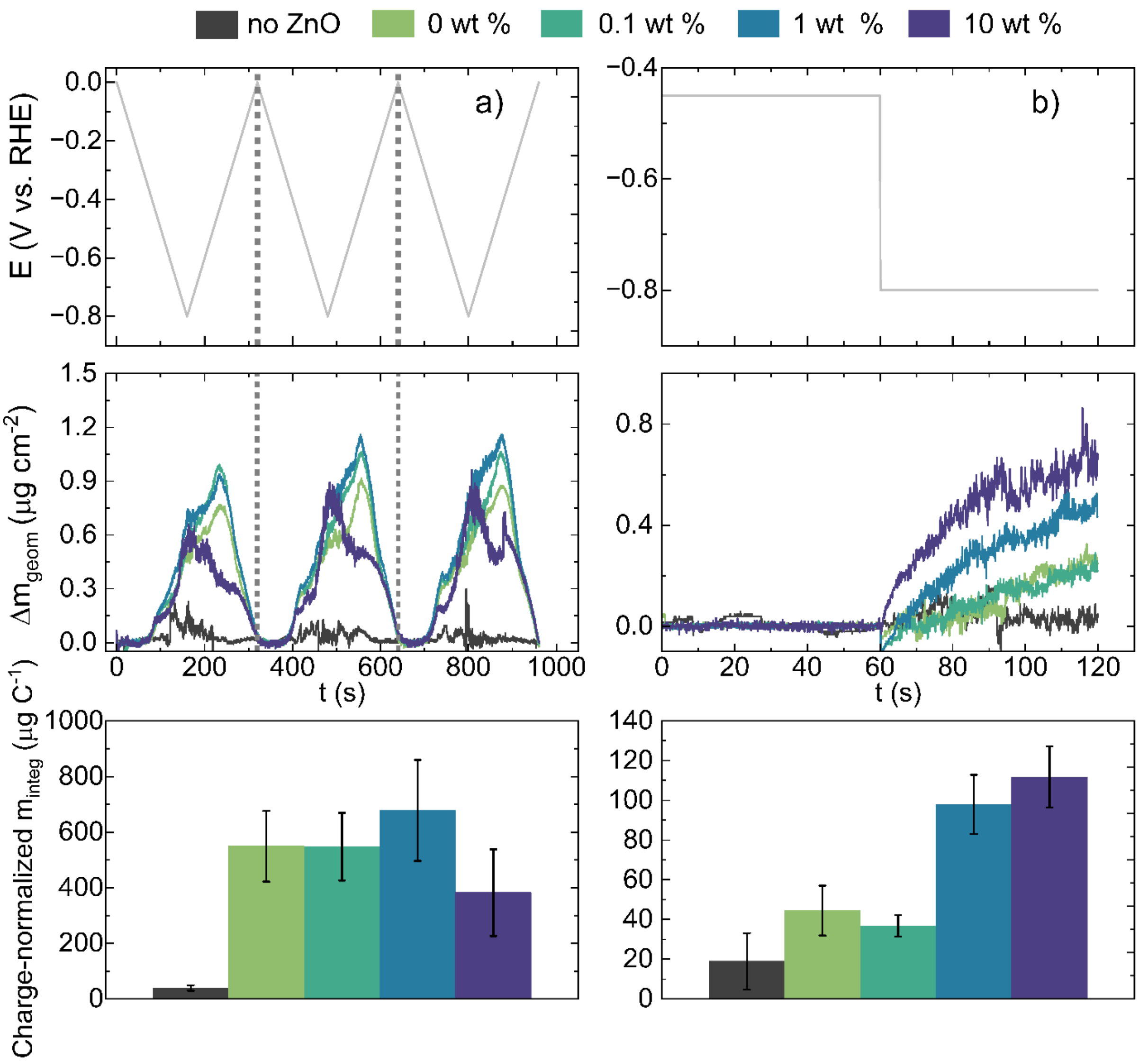

Supplementary Figure 22. Time-dependent mass gain profiles on pristine Au eQCM electrodes obtained in presence of dissolved zincate (1mM ZnO) with respect to PQ-2 contents in the KOH electrolyte (pH 13) during a) sequential cyclic voltammetry acquisition and b) on/off chronoamperometric holds. Bottom panels: charge-normalized mass gain (m_{integ}) stemming from the voltammograms/chronoamperograms.

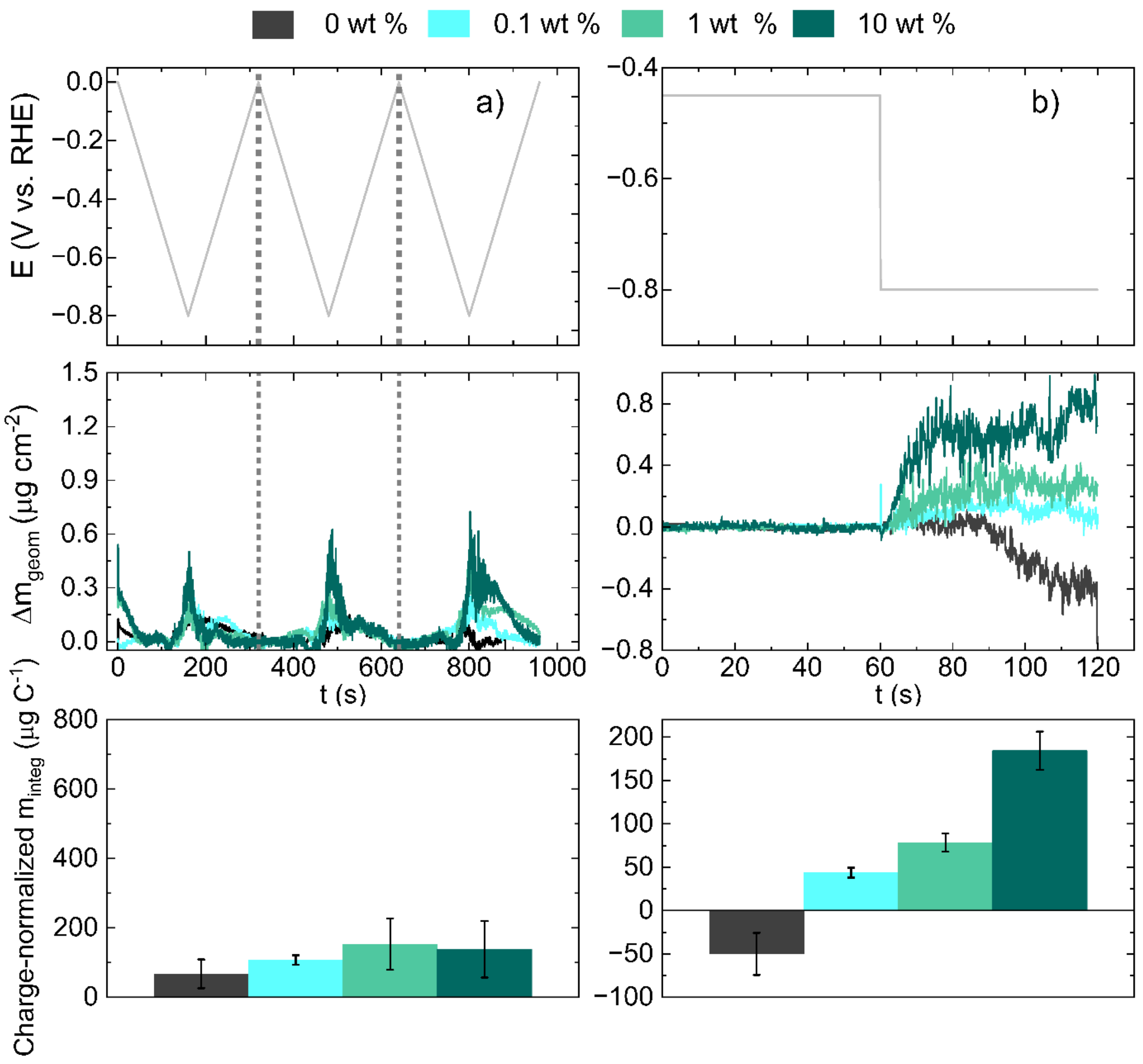

Supplementary Figure 23. Time-dependent mass gain profiles on pristine Au eQCM electrodes obtained in zincate-free KOH electrolyte (pH 13) with respect to PQ-2 contents during a) sequential cyclic voltammetry acquisition and b) on/off chronoamperometric holds. Bottom: charge-normalized mass gain (m_{integ}) stemming from voltammograms/ chronoamperograms.

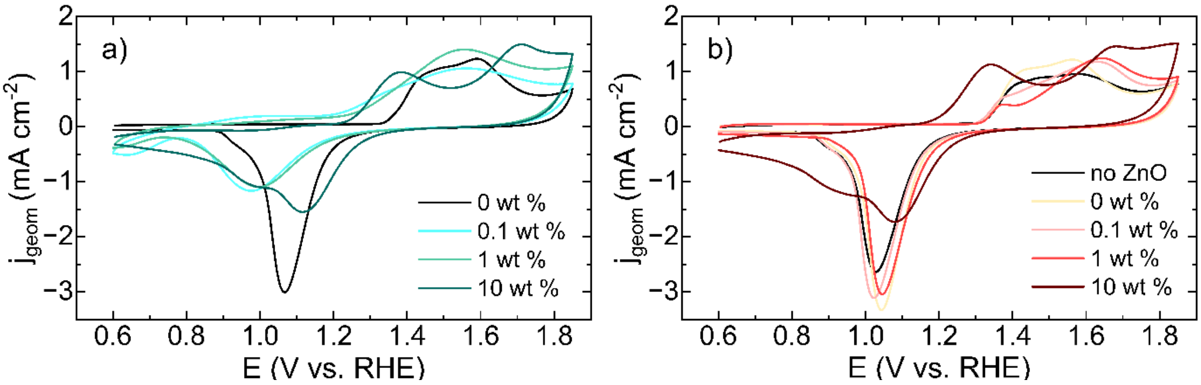

Supplementary Figure 24. Representative cyclic voltammograms obtained in Au-coated eQCM working electrodes after electrochemical cleaning in 3 % HNO_3 (50 CVs, 0.6 to 1.85 V_{RHE}) in absence (left) or in presence (right) of dissolved zincate (1mM ZnO) with respect to polyquaternium-2 (PQ-2) content (0, 0.1, 1 and 10 wt.%).

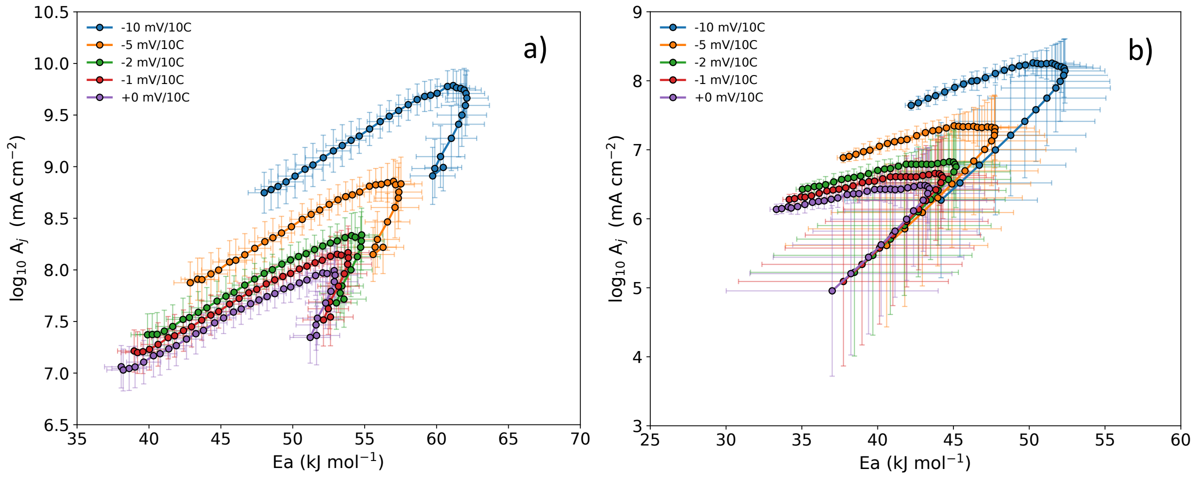

Supplementary Figure 25. The impact of temperature-dependent equilibrium shifts on the electrochemical Arrhenius activation parameters for to exemplary data sets. a) With ZnO and b) without ZnO in the electrolyte. Up to 2 mV/10˚C (5 mV total over a 25˚C temperature window) the variations in the apparent activation parameters are close to the error bars. However, for 5 mV/10˚C, let alone 10 mV/10˚C, the changes become substantial.

Supplementary References

1. Petersen, H. A. *et al.* On the Temperature Sensitivity of Electrochemical Reaction Thermodynamics. *ACS Phys. Chem Au* **3**, 241–251 (2023).
2. Lizée, M., Silva Olaya, A. R., Druce, J., Roldan Cuenya, B. & Oener, S. Z. Disentangling multistep kinetics by combining electrochemical Arrhenius analysis with micro-kinetic modelling. *Faraday Discussions* (2026).
3. Fingerhut, J. *et al.* Compensation effects between the apparent activation energy and pre-exponential factor in simple models of electrocatalytic hydrogen evolution. *Faraday Discussions* https://doi.org/10.1039/D5FD00163C (2026) doi:10.1039/D5FD00163C.
4. Wong, A. J.-W. *et al.* Entropy-enthalpy compensation in electrocatalytic rates. *ChemRxiv* 2026,.
